# Integrated bacterial transcriptome and host metabolome analysis reveals insights into “*Candidatus* Liberibacter asiaticus” population dynamics in the fruit pith of three citrus cultivars with different tolerance

**DOI:** 10.1128/spectrum.04052-23

**Published:** 2024-03-05

**Authors:** Yun Li, Ruifeng Ma, Chenying Gao, Ziyi Li, Yongqin Zheng, Fang Fang, Cheng Wang, Guohua Li, Xiaozhen Du, Changbao Xu, Meirong Xu, Rui Liu, Xiaoling Deng, Zheng Zheng

**Affiliations:** 1 National Key Laboratory of Green Pesticide, South China Agricultural University, Guangzhou, Guangdong, China; 2 Guangdong Province Key Laboratory of Microbial Signals and Disease Control, South China Agricultural University, Guangzhou, China; 3 Institute of Fruit Tree Research, Meizhou Academy of Agriculture and Forestry Sciences, Meizhou, Guangdong, China; 4 College of Horticulture, South China Agricultural University, Guangzhou, Guangdong, China; USDA - San Joaquin Valley Agricultural Sciences Center, Parlier, California, USA

**Keywords:** “*Candidatus *Liberibacter asiaticus”, fruit pith, dual RNA-Seq, bacterial transcriptome, metabolome, population dynamics

## Abstract

“*Candidatus* Liberibacter asiaticus” (CLas), the causal agent of citrus Huanglongbing (HLB), is able to multiply to a high abundance in citrus fruit pith. However, little is known about the biological processes and phytochemical substances that are vital for CLas colonization and growth in fruit pith. In this study, CLas-infected fruit pith of three citrus cultivars (“Shatangju” mandarin, “Guanxi” pomelo, and “Shatian” pomelo) exhibiting different tolerance to CLas were collected and used for dual RNA-Seq and untargeted metabolome analysis. Comparative transcriptome analysis found that the activation of the CLas noncyclic TCA pathway and pathogenic-related effectors could contribute to the colonization and growth of CLas in fruit pith. The pre-established Type 2 prophage in the CLas genome and the induction of its CRISPR/*cas* system could enhance the phage resistance of CLas and, in turn, facilitate CLas population growth in fruit pith. CLas infection caused the accumulation of amino acids that were correlated with tolerance to CLas. The accumulation of most sugars and organic acids in CLas-infected fruit pith, which could be due to the phloem blockage caused by CLas infection, was thought to be beneficial for CLas growth in localized phloem tissue. The higher levels of flavonoids and terpenoids in the fruit pith of CLas-tolerant cultivars, particularly those known for their antimicrobial properties, could hinder the growth of CLas. This study advances our understanding of CLas multiplication in fruit pith and offers novel insight into metabolites that could be responsible for tolerance to CLas or essential to CLas population growth.

**IMPORTANCE:**

Citrus Huanglongbing (HLB, also called citrus greening disease) is a highly destructive disease currently threatening citrus production worldwide. HLB is caused by an unculturable bacterial pathogen, “*Candidatus* Liberibacter asiaticus” (CLas). However, the mechanism of CLas colonization and growth in citrus hosts is poorly understood. In this study, we utilized the fruit pith tissue, which was able to maintain the CLas at a high abundance, as the materials for dual RNA-Seq and untargeted metabolome analysis, aiming to reveal the biological processes and phytochemical substances that are vital for CLas colonization and growth. We provided a genome-wide CLas transcriptome landscape in the fruit pith of three citrus cultivars with different tolerance and identified the important genes/pathways that contribute to CLas colonization and growth in the fruit pith. Metabolome profiling identified the key metabolites, which were mainly affected by CLas infection and influenced the population dynamic of CLas in fruit pith.

## INTRODUCTION

Citrus Huanglongbing (HLB) is the most destructive disease in citrus production worldwide. HLB is caused by the fastidious phloem-limited α-proteobacterium “*Candidatus* Liberibacter spp.,” mainly including three species, “*Ca*. L. asiaticus” (*C*Las), “*Ca*. L. africanus,” and “*Ca*. L. americanus.” Among the three species, CLas was the most widely distributed and caused the most significant economic losses to citrus production worldwide (1). Up to now, only CLas has been detected in HLB samples in China (2, 3). CLas was mainly vectored by Asian citrus psyllid (*Diaphorina citri* Kuwayama) between trees in the field (4). No effective control strategy is currently available for HLB, although the heat treatment has shown potential in reducing CLas titer in citrus seedlings (5). Nearly all commercially cultivated citrus varieties can be infected by CLas, although some were found to exhibit potential tolerance to CLas/HLB (6–8). Examination of host response of 30 different citrus genotypes identified the tolerance to HLB by Eureka lemon (*Citrus limon* Osbeck), Persian lime [*C. aurantifolia* (Christm.) Swingle] and Carrizo citrange [*C. sinensis* (L.) Osb. ×*Poncirus trifoliata* (L.) Raf.] (6). A recent study observed the HLB tolerance in *C. limon* and *C. maxima*, while *C. reticulata* Blanco and *C. sinensis* were more sensitive to HLB (7). Due to the current inability to culture CLas *in vitro*, research on CLas biology is mainly based on CLas-infected hosts (plant hosts or vector insects). However, the distribution of CLas was uneven in an infected citrus plant. Remarkably, a recent study reported a significantly higher abundance of CLas in fruit pith tissue than in leaf midribs and other infected tissues from the infected branch (9). The ability of fruit pith to support the multiplication of CLas to a relatively high level made it an excellent plant material for analyses of the biological processes involved in CLas growth and proliferation.

The *in-planta* genome-wide transcriptome profiling of plant pathogenic bacteria provides a comprehensive approach to gain insights into bacterial cellular changes during colonization and infection within plant host cells (10). However, the assessment of CLas transcriptome profiling *in planta* is challenging mainly due to its low abundance of bacterial RNA in total RNA extracted from infected host tissue. With the recent development of the dual RNA-Seq approach and the bacterial cell enrichment method, the transcriptome profiling of CLas in citrus or insect hosts became feasible (9, 11–13). CLas transcriptomic analysis based on bacterial cell isolation found that CLas genes involved in transcription/translation and resilience to host defense response were induced in citrus as compared to psyllid (12). Comparative transcriptional profiling of CLas in different types of host tissues revealed that CLas genes related to the transport system, stress response, replication, cell surface structure, and virulence were significantly up-regulated in leaf midribs as compared to fruit pith (9). Most recently, the genome-wide gene expression analyses of two CLas strains with different pathogenic phenotypes in periwinkle plants showed that the unique expression of genes involved in phage lytic activity and the difference in expression of virulence factor genes could be two major determinants responsible for virulence variation between two CLas strains (13). Analysis of the CLas transcriptome landscape in hosts has opened a new venue for CLas biology research. To date, studies in transcriptomic profiling of CLas on host are still in their infancy and have mainly focused on a limited number of citrus cultivars (mostly HLB-sensitive cultivars) or the infected insect vector to reveal the interaction between CLas and host (9, 12, 13). However, the biological functions and gene expression pathways that are important for CLas colonization and proliferation in citrus hosts with different tolerance are still lacking.

In addition to HLB transcriptome research, metabolomics has also been utilized to evaluate metabolite changes in citrus leaf after infection with CLas (14–16), nutritional needs for CLas growth (17), metabolites related to HLB tolerance (18–21), effects of CLas infection on juice quality (22–25), and abnormal color of citrus peel caused by CLas infection (26). Research on citrus metabolic response against HLB found that the secondary metabolites known for their antibacterial activity were correlated with citrus tolerance against CLas (27). In addition to citrus hosts, periwinkle was found to be a more reliable experimental host plant for CLas as its advantages in supporting the rapid establishment and greater multiplication of CLas (28, 29). The comparative metabolomic profiling of periwinkle (*Catharanthus roseus*) and sweet orange “Valencia” (*Citrus sinensis*) in response to CLas infection was performed to identify the potential major nutritional needs for CLas growth within leaf midrib (17). In addition to periwinkle, citrus fruit pith, which was able to support the multiplication of CLas to relatively high levels (9), can also be used as an ideal host tissue for metabolomics analyses of CLas-host interactions and nutritional needs for CLas growth. However, except of a single report of metabolite profiling of leaf midrib and fruit pith (30), little is known about the fundamental metabolic shift in fruit pith initiated by CLas colonization, particularly in citrus cultivars with different tolerance to CLas.

Here, we performed the dual RNA-Seq-based bacterial transcriptome analyses and untargeted metabolome analyses of healthy and CLas-infected fruit pith from three commercial cultivars with different tolerance to CLas, including two *C. maxima* cultivars (“Shatian” pomelo, *C. maxima* cv. “Shatian Yu,” and “Guanxi” pomelo, *C. maxima* cv. “Guanxi Yu”) and a *C. reticulata* Blanco cultivar (“Shatangju mandarin,” *C. reticulata* Blanco cv. “Shatangju”). Comparative quantification analyses showed that the fruit pith of “Shatangju” mandarin could be more favorable for CLas growth by containing a relatively higher CLas concentration than those identified in two pomelo cultivars. Genome-wide gene expression profiling of CLas was further performed to reveal the gene expression related to the colonization and growth of CLas in the fruit pith of three cultivars. In addition, comparative metabolome analyses of CLas-infected and healthy fruit pith were also performed to investigate the metabolic change in the host response to CLas infection among three cultivars. The alterations in metabolites responsible for the difference in CLas concentration in fruit pith among three cultivars were analyzed. Our result will improve our understanding of gene expression patterns and biological processes related to CLas multiplication and provide new insight into the CLas-host interaction in fruit pith.

## RESULTS

### Quantification of CLas and its associated phage in fruit pith of three cultivars

Quantitative analyses showed that the average concentration of CLas in fruit pith of “Shatangju” mandarin (630,595 ± 103,264 cells/ng of total DNA) was significantly higher than those observed in fruit pith of “Guanxi” pomelo (35,469 ± 10,460 cells/ng of total DNA) and “Shatian” pomelo (10,946 ± 1,328 cells/ng of total DNA) (*P* < 0.05) (Table 1). Based on the difference in CLas population in the fruit pith of three cultivars, as well as the previously reported greater tolerance to CLas in “Shatian” pomelo as compared to “Shatangju” mandarin (7), the fruit pith of “Shatangju” mandarin should be more favorable for CLas growth as compared to “Guanxi” pomelo and “Shatian” pomelo.

**TABLE 1:** Quantification of “*Candidatus* Liberibacter asiaticus” (CLas) and the associated phage from fruit pith of three cultivars

No.	Cultivar	CLas cells/ng of total DNA^*a*^	Copy number of phages per CLas cell^*a*^		
			Type 1	Type 2	Type 3
1	“Guanxi” pomelo	35,469 ± 10,460 b	NA	13.8 ± 1.5	NA
2	“Shatian” pomelo	10,946 ± 1,328 c	0.1 ± 0.0	1.0 ± 0.4	0.1 ± 0.0
3	“Shatangju” mandarin	630,595 ± 103,264 a	NA	1.3 ± 0.4	NA

^*a*^ NA, not applicable. The different letters represent significant differences by single-factor analysis of variance (Duncan’s multiple-range test) at a 95% (*P* < 0.05) confidence interval.

Phage typing result showed that all CLas samples from “Shatian” pomelo contained three types of phage (Type 1, Type 2, and Type 3), while CLas samples from “Guanxi” pomelo and “Shatangju” mandarin only contained Type 2 phage (Table 1). Estimating of phage copy number showed that CLas samples from “Guanxi” pomelo contained multiple copies of Type 2 (13.8 copies per CLas cells), while CLas samples from “Shatangju” mandarin contained a nearly single copy of Type 2 phage (1.3 copies per CLas cell). It should be noted that CLas samples from “Shatian” pomelo contained a relatively low density of Type 1 (0.1 copy per CLas cell) and Type 3 phage (0.1 copy per CLas cell) but a single copy of Type 2 phage (1.0 copy per CLas cell) (Table 1).

### Transcriptome data analysis with CLas-infected fruit pith

A total of nine dual RNA-Seq libraries for RNA samples extracted from CLas-infected and healthy fruit pith tissue of three citrus cultivars were constructed and sequenced in this study. The Illumina HiSeq platform generated a sequencing depth of 130–144 million 150 bp paired-end reads per library with Q20 >95 (Table S1). A high correlation was observed among three biological replicates with Pearson’s correlation coefficient over 0.96, indicating the high repeatability and quality of raw RNA-Seq data generated in this study (Table S2). The total number of reads that mapped to CLas and the associated phage reference sequences ranged from 377,773 to 683,562, counting from 1.21% to 1.94% of total reads, among RNA-Seq data generated from CLas-infected fruit pith of three cultivars (Table S1). Since CLas present at significantly higher concentration in the fruit pith of “Shatangju” mandarin than in fruit pith of “Guanxi” pomelo and “Shatian” pomelo, the CLas RNA-Seq data of “Shatangju” mandarin was used as control to identified CLas differentially expressed genes (DEGs) in “Guanxi” pomelo and “Shatian” pomelo. The DEGs and highly expressed genes related to CLas adaption and multiplication in fruit pith were described in detail in the following section.

### Differential expression of CLas chromosomal genes in fruit pith

**Fig 1:**
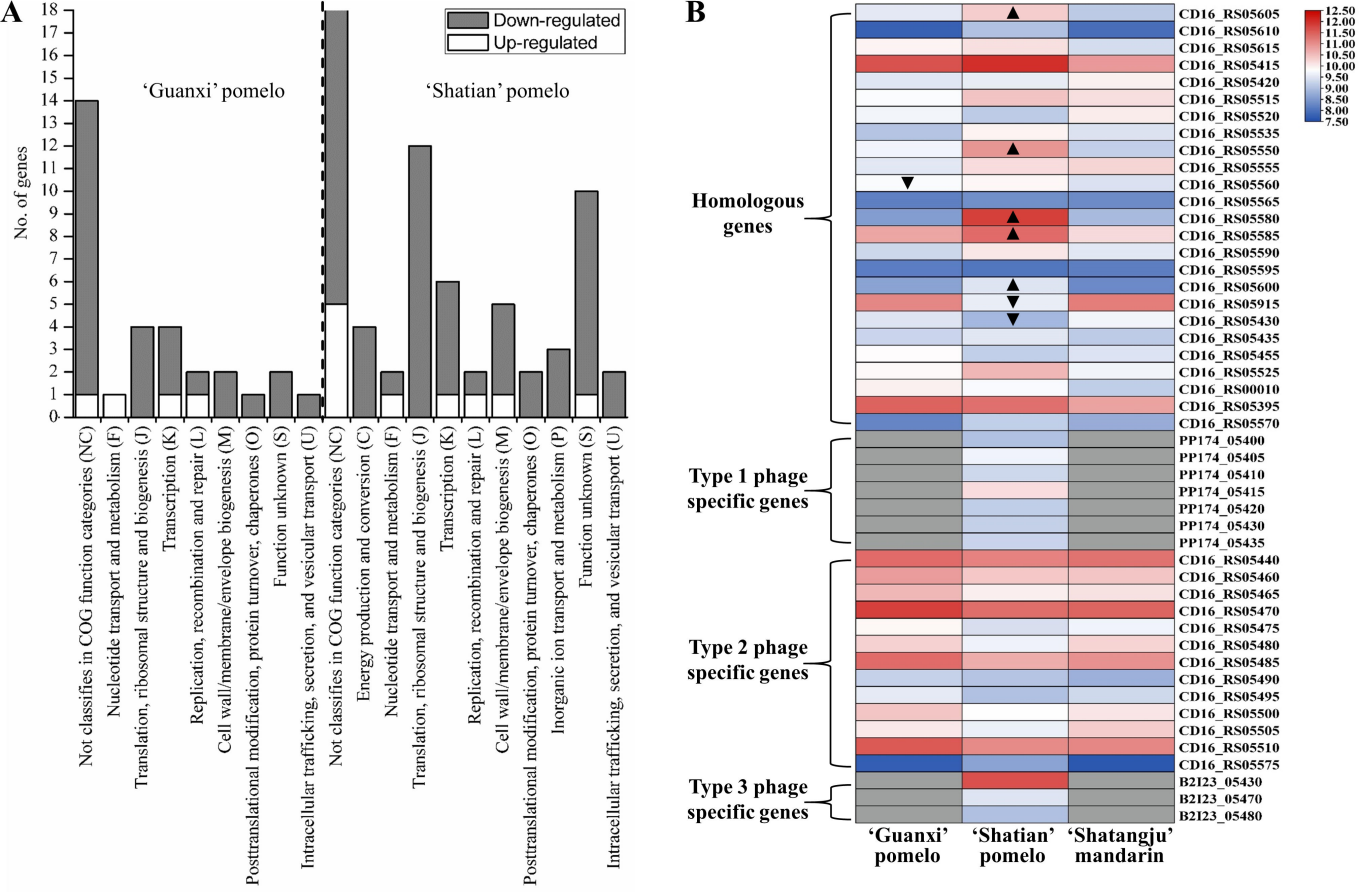
Functional classification and heatmap of DEGs of “*Candidatus* Liberibacter asiaticus” (CLas) in fruit pith of three cultivars. (**A**) Function classification of CLas chromosomal DEGs. (**B**) Heatmap showing the gene expression of selected phage/prophage genes (TPM value >500). ▲, up-regulated gene (compared to “Shatangju” mandarin). ▼, down-regulated gene (compared to “Shatangju” mandarin). The gray box indicates no TPM value due to no homologous gene identified in the corresponding CLas strain.

Compared to RNA-Seq data from “Shatangju” mandarin, a total of 30 DEGs and 68 DEGs from the CLas chromosomal region were identified in CLas strain from “Guanxi” pomelo and “Shatian” pomelo, respectively (Table S3). Particularly, most of the CLas chromosomal DEGs were repressed in “Guanxi” pomelo and “Shatian” pomelo, including 27 in “Guanxi” pomelo and 59 in “Shatian” pomelo (Table S3). Functional classification showed that the down-regulated CLas chromosomal DEGs in “Guanxi” pomelo and “Shatian” pomelo were mainly involved in transcription, translation, ribosomal structure, and biogenesis (Fig. 1A). Moreover, CLas chromosomal DEGs involved in energy production and conversion, inorganic ion transport and metabolism were also down-regulated in the fruit pith of “Shatian” pomelo as compared to “Shatangju” mandarin (Fig. 1A). Among down-regulated CLas chromosomal DEGs, three key enzymes related to CLas TCA cycle (tricarboxylic acid cycle), the isocitrate dehydrogenase (CD16_RS03985), malate dehydrogenase (CD16_RS04710), and fumarate hydratase (CD16_RS00320), were repressed in both “Guanxi” pomelo and “Shatian” pomelo as compared to “Shatangju” mandarin (Table S3). Four DEGs with the function of pathogenic effector or virulence factor, including CD16_RS02255, CD16_RS04385, CD16_RS05110, and CD16_RS05720, were down-regulated in “Guanxi” pomelo and “Shatian” pomelo (Table S3). Only three DEGs, including a ribonucleotide-diphosphate reductase subunit beta (CD16_RS04295), DEAD/DEAH box helicase (CD16_RS05750), and a hypothetical protein gene (CD16_RS00025), were up-regulated in fruit pith of “Guanxi” pomelo and “Shatian” pomelo compared to “Shatangju” mandarin (Table S3).

### Differential expression of phage/prophage genes in fruit pith

Sequence comparison of three types of CLas-associated phage (Type 1: P-YNBC-1, Type 2: P-A4-2, and Type 3: P-JXGC-3) identified a total of 32 homologous genes, 13 Type 1 phage-specific genes, 15 Type 2 phage-specific genes, and 11 Type 3 phage-specific genes (Table S4). Of 32 homologous genes, only three DEGs and nine DEGs identified in the fruit pith of “Guanxi” pomelo and “Shatian” pomelo as compared to “Shatangju” mandarin, respectively (Table S4). Among nine DEGs identified in the fruit pith of “Shatian” pomelo, six were up-regulated and three were down-regulated. Interestingly, four up-regulated DEGs (CD16_RS05600, 05605, 05580, and 05585) in the fruit pith of “Shatian” pomelo were belong to a previously reported CRISPR/*cas* system located in CLas prophage region (31). Remarkably, other genes involved in CLas CRISPR/*cas* system, including CD16_RS05610, 05615, 05410, 05590, 05570, and 05575, also showed the higher expression levels in fruit pith of “Shatian” pomelo than those observed in “Guanxi” pomelo and “Shatangju” mandarin, although the difference was not significant (Fig. 1B; Table S4). Among others, phage homologous genes worth highlighting were a guanylate kinase (CD16_RS05415) and a DNA polymerase (CD16_RS05395), which both exhibited relatively high expression levels (TPM value >1500) in the fruit pith of all three cultivars (Fig. 1B; Table S4).

In addition to homologous genes among three phages, the expression level of phage type-specific genes was also analyzed. Overall, most of the Type 2 phage-specific genes were highly expressed (TPM >500), while most of the Type 1 and Type 3 phage-specific were in a low expression level (TPM <500) in the fruit pith tissue (Table S4). Among Type 1 phage-specific genes, genes involved in phage lytic activity, phage capsid structure, and assembly were uniquely expressed but with a low expression level in the fruit pith of “Shatian” pomelo (Table S4). For Type 2 phage-specific genes, no significant difference was observed in expression level among CLas samples from three cultivars (Fig. 1B; Table S4). The highly expressed Type 2 phage-specific genes mainly included genes encoded head protein (CD16_RS05465), tail protein (CD16_RS05485, CD16_RS05510), integrase (CD16_RS05475), protease (CD16_RS05500), exonuclease (CD16_RS05480), phage-related glutathione peroxidase (CD16_RS05505), and phage-related proteins (CD16_RS05440, CD16_RS05460, CD16_RS05470) (Fig. 1B; Table S4). For Type 3 phage-specific genes, only the signal recognition particle receptor FtsY alpha subunit gene (B2I23_05430) showed a relatively high expression level (TPM = 3,256) in CLas samples from “Shatian” pomelo (Fig. 1B; Table S4).

### CLas genes with significant high expression in fruit pith

**Fig 2:**
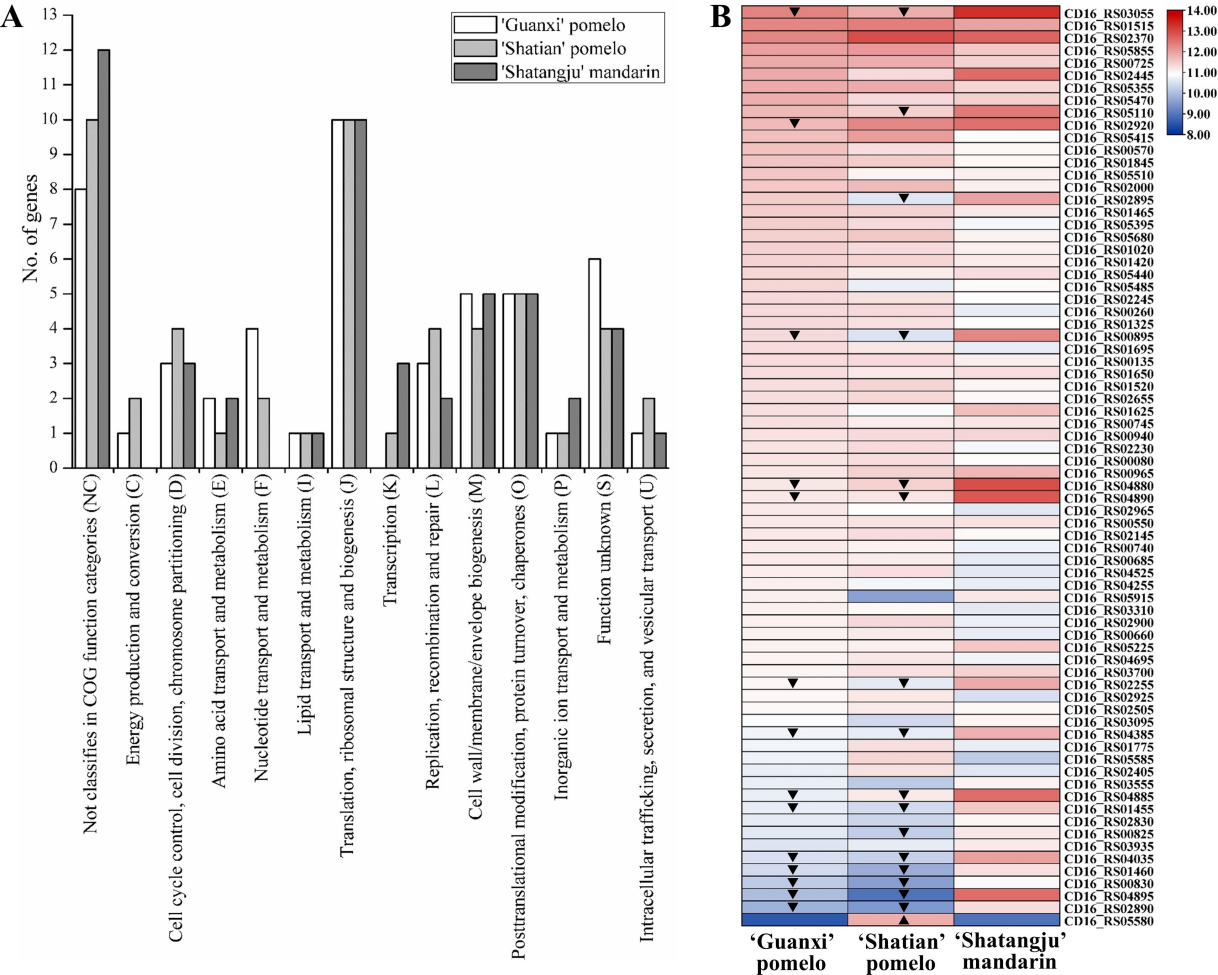
Functional classification (**A**) and heatmap (**B**) of top 50 highly expressed genes of “*Candidatus* Liberibacter asiaticus” (CLas) in fruit pith of three cultivars. ▲, up-regulated gene (compared to “Shatangju” mandarin). ▼, down-regulated gene (compared to “Shatangju” mandarin).

To analyze which CLas gene functions may facilitate the adaptation and proliferation of CLas, the highly expressed CLas genes in the fruit pith of three cultivars with different tolerance were compared. The top 50 highest expressed genes from three cultivars were selected and analyzed (Fig. 2; Table S5). Functional enrichment analyses found that a large number of genes were related to housekeeping activities, including translation, ribosomal structure and biogenesis, posttranslational modification, protein turnover and chaperones, cell wall, membrane, and envelope biogenesis (Fig. 2A). CLas genes with functions related to transport and metabolism of amino acids, nucleotide, lipid, and inorganic ion were also enriched in fruit pith of three cultivars, including 4-hydroxy-tetrahydrodipicolinate synthase (CD16_RS00745), phosphoserine transaminase (CD16_RS00135), UMP kinase (CD16_RS02145), guanylate kinase (CD16_RS05415), thymidylate synthase (CD16_RS04255), adenylosuccinate synthase (CD16_RS02965), type II toxin-antitoxin system RatA family toxin (CD16_RS02655), ferritin (CD16_RS02445), and superoxide dismutase (CD16_RS03555) (Fig. 2B; Table S5). It was also found that CLas genes involved in DNA replication and repair, cell cycle control, division, and chromosome partitioning were highly expressed in three cultivars (Fig. 2B; Table S5). Among others, genes worth highlighting was Flp family type IVb pilin (CD16_RS02370), which exhibited relatively high expression level (TPM >5,000) in the fruit pith of all three cultivars (Table S5). In addition, four genes with functions as pathogenic effectors or virulence factor were highly expressed in three cultivars, including CD16_RS02255, CD16_RS04385, CD16_RS05110, and CD16_RS05225 (Table S5).

### Multivariate analyses of metabolic compounds in CLas-infected and healthy fruit pith of three cultivars

**Fig 3:**
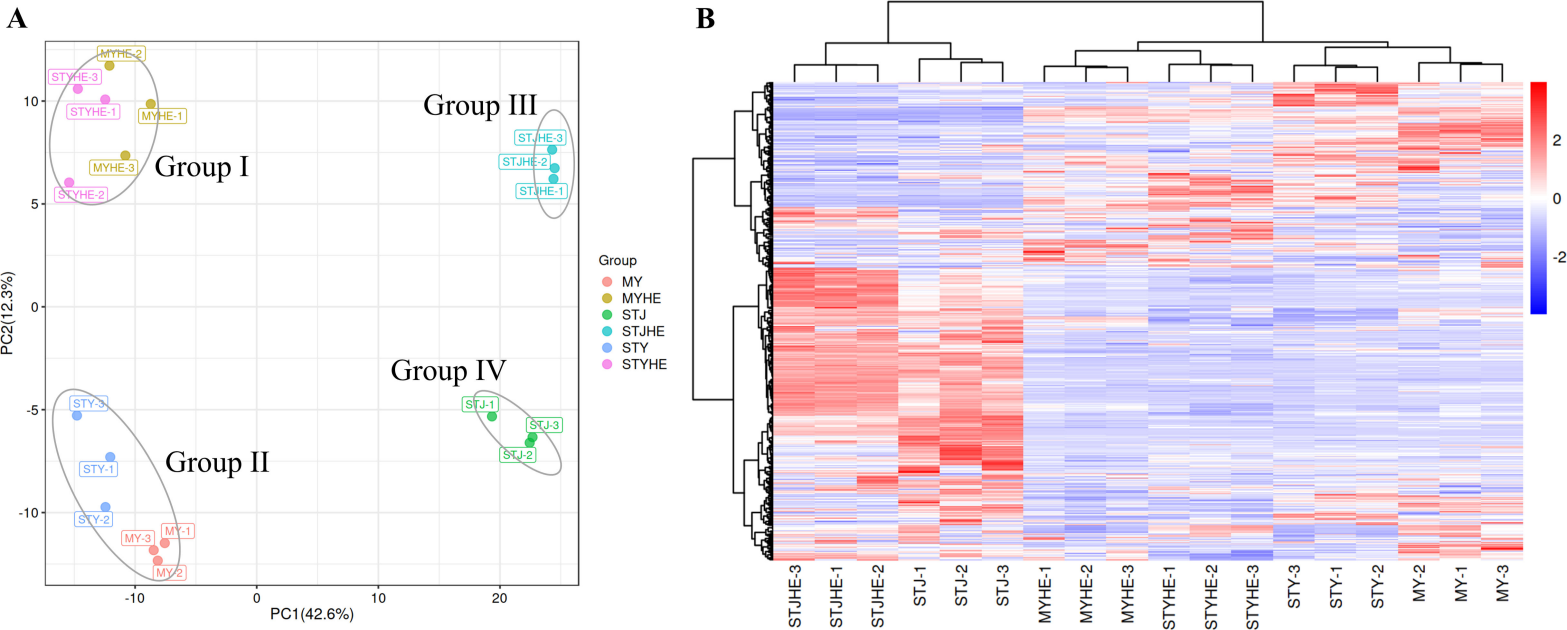
The principal component analysis and clustering heatmap of total sample metabolites from “*Candidatus* Liberibacter asiaticus”-infected and healthy fruit pith tissue of three cultivars. MY, CLas-infected “Guanxi” pomelo. MYHe, Healthy “Guanxi” pomelo. STY, CLas-infected “Shatian” pomelo. STYHe, Healthy “Shatian” pomelo. STJ, CLas-infected “Shatangju” mandarin. STJHe, Healthy “Shatangju” mandarin.

Comparative metabolic profiling between CLas-infected and healthy fruit pith from three cultivars was performed to analyze the metabolic changes in fruit pith in response to CLas infection. The PCA scatter plot showed that PC1 and PC2 explained 54.9% of the total difference, in which PC1 accounted for 42.6%, and PC2 accounted for 12.3% of the total variation of normalized LC-MS data based on CLas-infected and healthy fruit pith tissue of three cultivars (Fig. 3A). Three replicate samples from the same group were closely clustered and an obvious distinction between CLas-infected and healthy fruit pith of three cultivars were also observed by PLS-DA and OPLS-DA (Fig. S1). Four separated clusters were identified in the PCA scatter plot, including Group I, consisting of samples from healthy fruit pith tissue of “Guanxi” pomelo and “Shatian” pomelo; Group II, consisting of samples from CLas-infected fruit pith tissue of “Guanxi” pomelo and “Shatian” pomelo; Group III, consisting of samples from healthy fruit pith tissue of “Shatangju” mandarin; and Group IV, consisting of samples from CLas-infected fruit pith tissue of “Shatangju” mandarin (Fig. 3A). The cluster result showed that the metabolites identified in healthy or CLas-infected fruit pith tissue of two pomelo cultivars were much more similar as compared to “Shatangju” mandarin fruit pith (Fig. 3A). In addition, the heatmap of metabolites showed the similar cluster result as PCA/PLS-DA/OPLS-DA cluster result of healthy and CLas-infected samples from three cultivars (Fig. 3B).

### Comparative metabolic analysis of CLas-infected and healthy fruit pith of three cultivars

**Fig 4:**
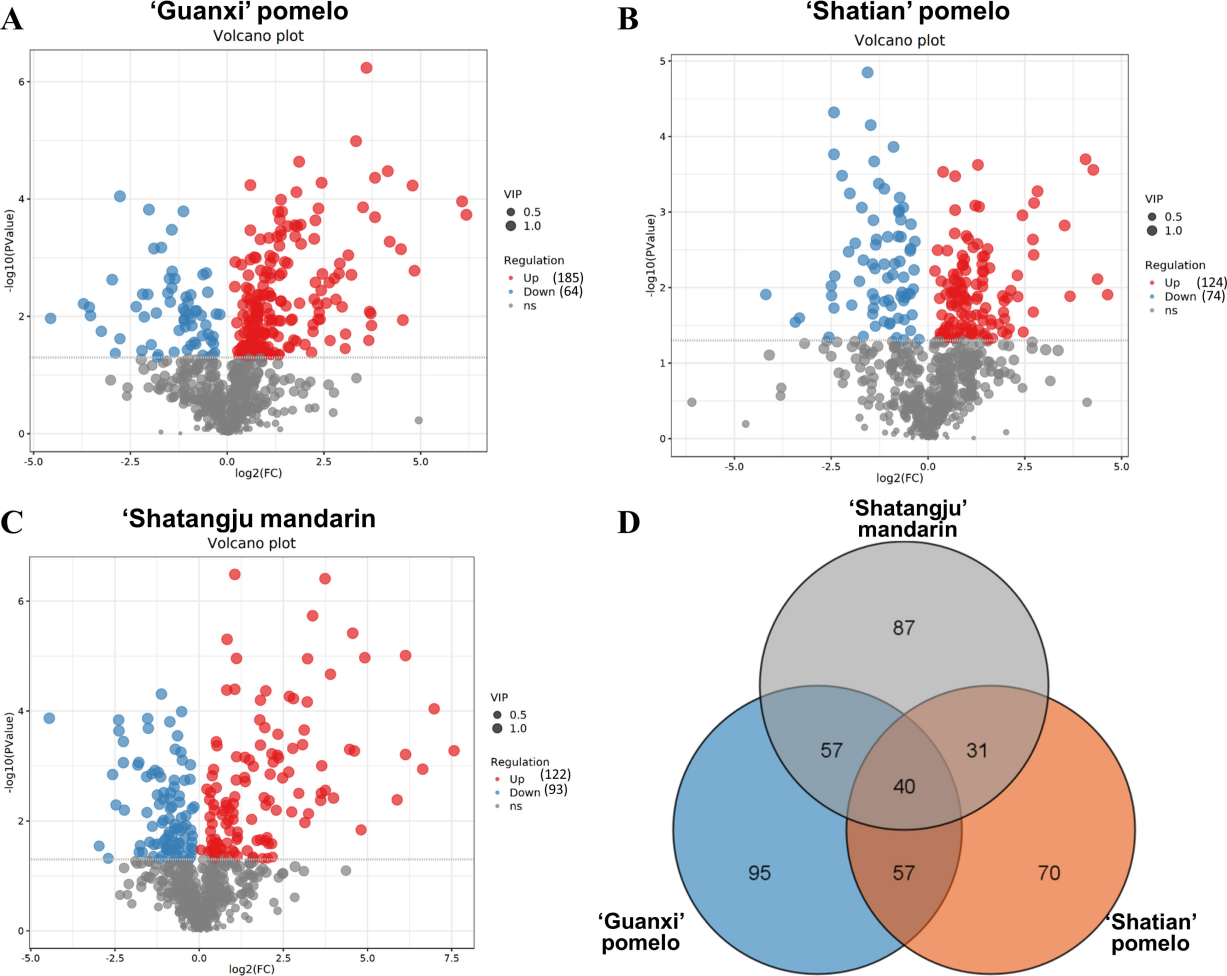
Volcano plot and Venn diagram of differential metabolites between “*Candidatus* Liberibacter asiaticus”-infected and healthy fruit pith tissue of three cultivars. (**A**) “Guanxi” pomelo. (**B**) “Shatian” pomelo. (**C**) “Shatangju” mandarin. (**D**) Venn cluster of differential metabolites among three groups.

Comparative metabolic analyses identified a total of 668 with differential abundance in CLas-infected fruit pith as compared to healthy fruit pith of three cultivars, including 249 in “Guanxi” pomelo, 198 in “Shatian” pomelo, and 215 in “Shatangju” mandarin (Fig. 4). Of 249 altered metabolites in CLas-infected fruit pith of “Guanxi” pomelo, 185 were increased and 64 were depleted as compared to the healthy control (Fig. 4). In “Shatian” pomelo, 124 accumulated metabolites and 74 decreased metabolites were identified in CLas-infected fruit pith compared to healthy fruit pith (Fig. 4). Among 215 altered compounds in CLas-infected fruit pith of “Shatangju” mandarin, 122 were accumulated and 93 were depleted (Fig. 4). Venn diagram of differentially abundant compounds identified 40 common metabolites among three cultivars (Fig. 4). A total of 95, 70, and 87 differentially abundant compounds were uniquely found in CLas-infected “Guanxi” pomelo, “Shatian” pomelo, and “Shatangju” mandarin as compared to the corresponding healthy control, respectively (Fig. 4).

**Fig 5:**
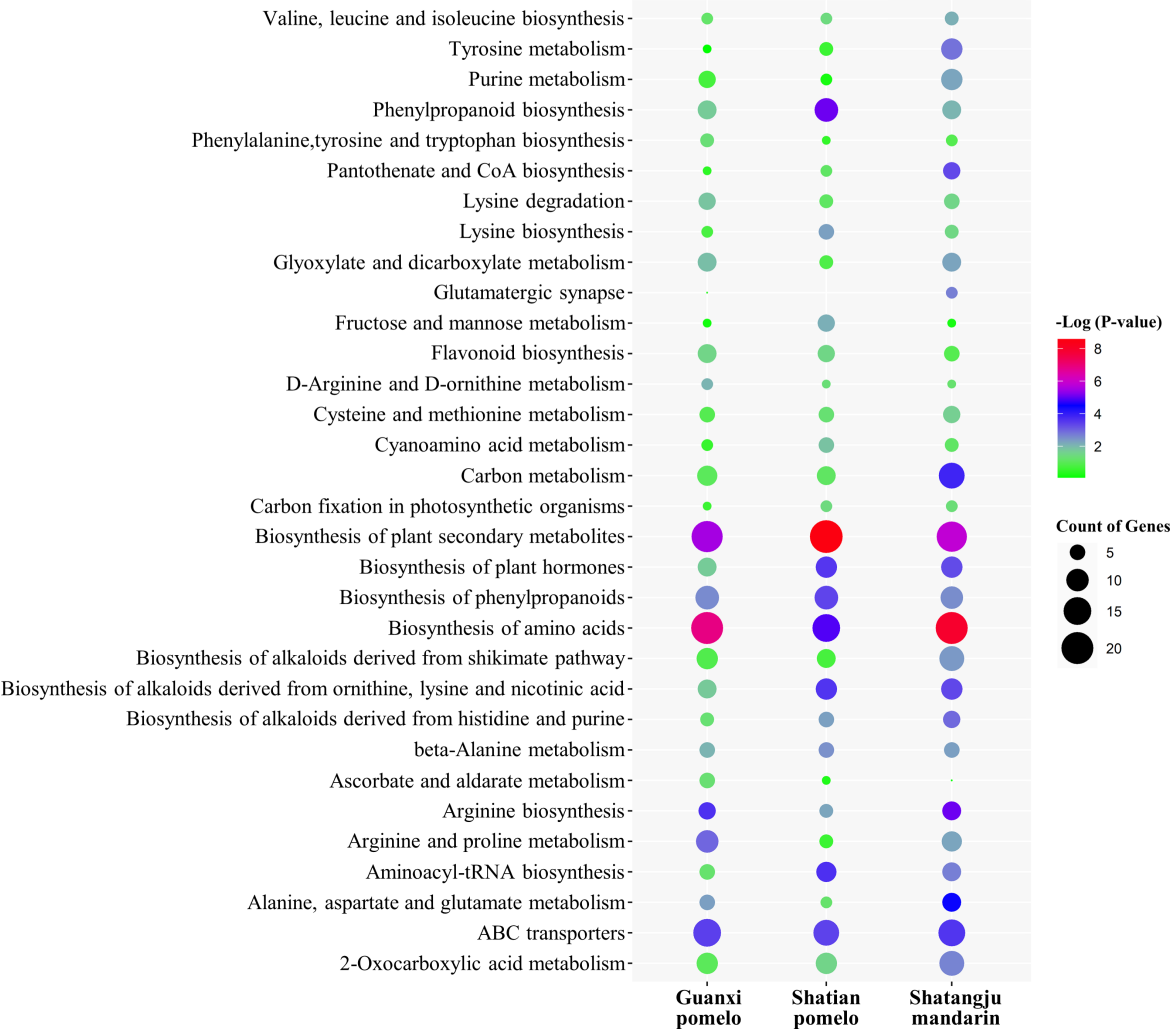
KEGG enrichment analysis of metabolites differently abundant between “*Candidatus* Liberibacter asiaticus”-infected and healthy fruit pith of three cultivars. The vertical coordinates represent the enriched pathways. The size of each point represents the number of differential metabolites in the pathway and the color of the point represents the *P*-value.

Pathway analysis of differentially abundant metabolites found that the biosynthesis of plant secondary metabolites, amino acids, phenylpropanoids, plant hormones, alkaloids, arginine and proline metabolism, ABC transporters, 2-Oxocarboxylic acid metabolism, and carbon metabolism was mainly altered in CLas-infected fruit pith of three cultivars as compared to the healthy fruit pith (Fig. 5). To further analyze the metabolites changes caused by CLas infection and assess the phytochemical substances related to the growth and proliferation of CLas in fruit pith, the primary metabolites (amino acids, sugars, and organic acids) and secondary metabolites (flavonoids and terpenoids) were compared between healthy and CLas-infected fruit pith tissue from three cultivars.

### Alteration of amino acids, sugars, and organic acids in fruit pith infected by CLas

**Fig 6:**
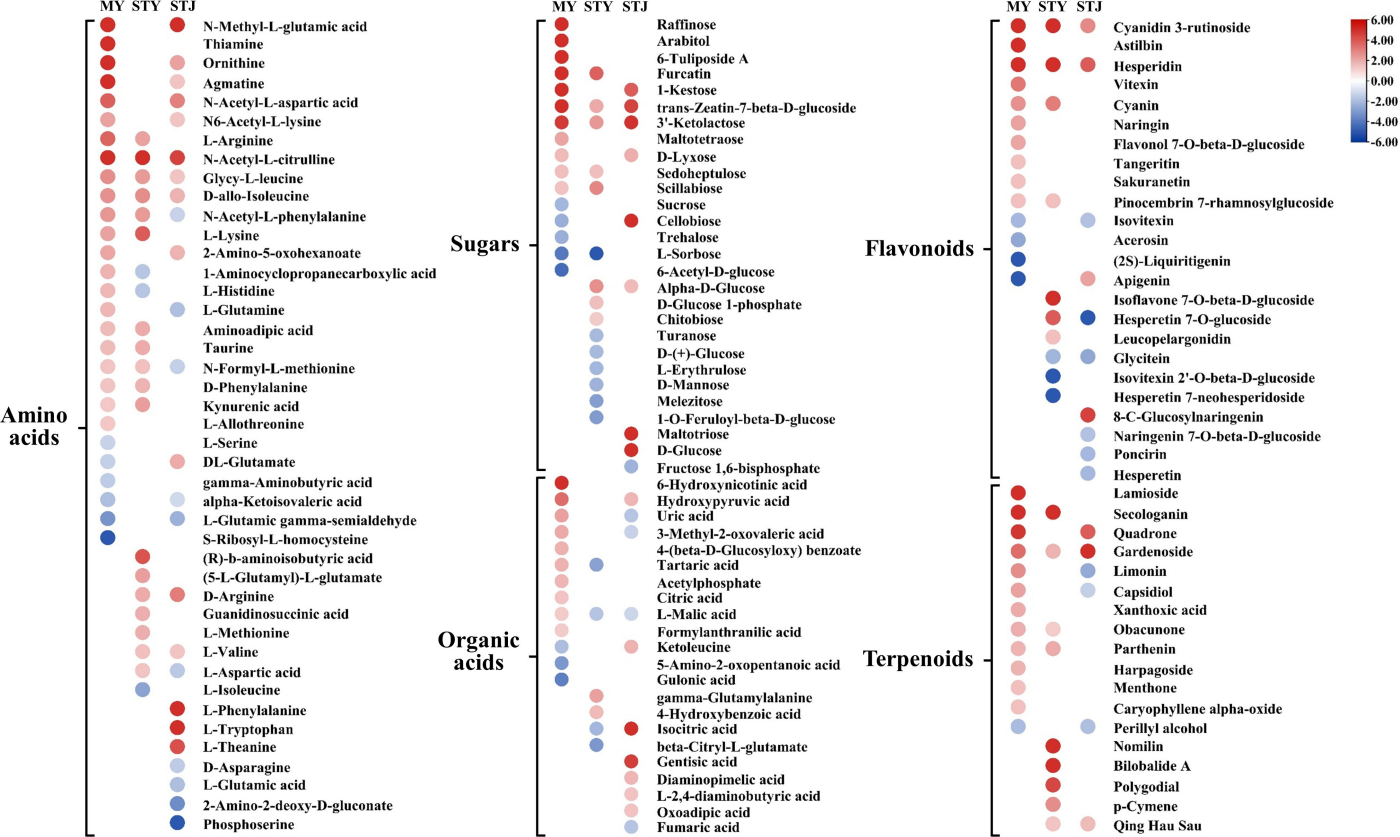
Heatmap of metabolites differently abundant between “*Candidatus* Liberibacter asiaticus” (CLas)-infected and healthy fruit pith of three cultivars. MY, CLas-infected “Guanxi” pomelo. STY, CLas-infected “Shatian” pomelo. STJ, CLas-infected “Shatangju” mandarin.

The amino acids account for the majority of differential abundance compounds identified between CLas-infected and healthy fruit pith tissue from three citrus cultivars. A total of 43 amino acids showed different abundance between CLas-infected and healthy fruit piths (Fig. 6). Overall, most of the amino acids showed increased levels in CLas-infected fruit pith as compared to healthy fruit pith in three cultivars (Fig. 6; Table S6). Particularly, an increased number of amino acids with accumulated levels was observed in CLas-infected fruit pith of “Guanxi” pomelo and “Shatian” pomelo as compared to “Shatangju” mandarin (Fig. 6). A total of 22 and 18 amino acids showed higher level in CLas-infected fruit pith of “Guanxi” pomelo and “Shatian” pomelo than healthy control, respectively (Fig. 6). Eight accumulated amino acids were only identified in CLas-infected fruit pith of “Guanxi” pomelo and “Shatian” pomelo as compared to the CLas-infected fruit pith of “Shatangju” mandarin, including L-arginine, N-Acetyl-L-phenylalanine, L-lysine, aminoadipic acid, taurine, N-formyl-L-methionine, D-Phenylalanine, and kynurenic acid (Fig. 6). A total of 15 amino acids showed increased levels in CLas-infected fruit pith of “Shatangju” mandarin compared to the healthy control and four were only accumulated in CLas-infected fruit pith of “Shatangju” mandarin, including DL-Glutamate (2.0-fold), L-Phenylalanine (9.3-fold), L-Tryptophan (5.6-fold), and L-Theanine (4.2-fold) (Fig. 6; Table S7). By contrast, eight amino acids only decreased depleted in CLas-infected fruit pith of “Shatangju” mandarin, including N-Acetyl-L-phenylalanine, L-glutamine, N-formyl-L-methionine, L-aspartic acid, D-asparagine, L-glutamic acid, 2-Amino-2-deoxy-D-gluconate, and phosphoserine (Fig. 6).

Comparative metabolites profiling identified 28 altered sugars between the CLas-infected and healthy fruit piths of three cultivars (Fig. 6). Most of altered sugars showed increased levels in CLas-infected fruit pith of three cultivars, including 11 in “Guanxi” pomelo, eight in “Shatian” pomelo, and eight in “Shatangju” mandarin (Fig. 6). Among accumulated sugars, eight were accumulated in the CLas-infected fruit pith of at least two cultivars as compared to the healthy control, including furcatin, 1-kestose, trans-zeatin-7-beta-D-glucoside, 3′-ketolactose, D-lyxose, sedoheptulose, scillabiose, and alpha-D-glucose (Table S8). Three sugars, cellobiose (7.8-fold), maltotriose (126.1-fold), and D-glucose (5.0-fold), were only accumulated in CLas-infected fruit pith of “Shatangju” mandarin (Fig. 6; Table S7). In addition, six sugars (turanose, D-(+)-glucose, L-erythrulose, D-mannose, melezitose and 1-O-Feruloyl-beta-D-glucose) only showed decreased levels in CLas-infected “Shatian” pomelo and four sugars (sucrose, cellobiose, trehalose, and 6-Acetyl-D-glucose) only decreased in “Guanxi” pomelo (Fig. 6). The L-Sorbose were depleted in both CLas-infected fruit pith of two pomelo cultivars. The level of fructose 1,6-bisphosphate was only decreased in CLas-infected fruit pith of “Shatangju” mandarin as compared to healthy control (Fig. 6).

Comparative metabolites profile identified a total of 22 organic acids differently abundant between CLas-infected fruit pith and healthy fruit pith of three cultivars (Fig. 6). The number of altered organic acids varied among the three cultivars. Compared to “Shatian” pomelo, a higher number of organic acids with increased levels were identified in CLas-infected fruit pith of “Guanxi” pomelo and “Shatangju” mandarin (Fig. 6). Ten and seven organic acids showed higher levels in CLas-infected fruit pith of “Guanxi” pomelo and “Shatangju” mandarin than healthy control, respectively (Fig. 6). Six organic acids were only accumulated in CLas-infected fruit pith of “Shatangju” mandarin (Fig. 6). Particularly, the isocitric acid showed 190.5-fold higher in CLas-infected fruit pith of “Shatangju” mandarin (Fig. 6; Table S8). By contrast, three organic acids (uric acid, 3-Methyl-2-oxovaleric acid, and fumaric acid) were only decreased in CLas-infected “Shatangju” mandarin and L-malic acid was depleted in CLas-infected fruit pith of “Shatian” pomelo and “Shatangju” mandarin (Fig. 6).

### Changes in flavonoids and terpenoids in fruit pith infected by CLas

A total of 24 altered flavonoids were identified between CLas-infected fruit pith and healthy fruit pith of three cultivars (Fig. 6). Overall, compared to “Shatangju” mandarin, a higher number of flavonoids with increased levels was observed in CLas-infected fruit pith of “Guanxi” pomelo and “Shatian” pomelo (Fig. 6). Particularly, among 14 altered flavonoids in CLas-infected fruit pith of “Guanxi” pomelo, 10 were increased and only four were reduced (Fig. 6). It was found that cyanidin 3-rutinoside and hesperidin were accumulated in CLas-infected fruit pith of all three cultivars (Fig. 6). However, the increased fold of both cyanidin 3-rutinoside and hesperidin was significantly higher in fruit pith of “Guanxi” pomelo and “Shatian” pomelo than in “Shatangju” mandarin (Fig. 6). Two flavonoids, cyanin and pinocembrin 7-rhamnosylglucoside, were only showed higher levels in CLas-infected fruit pith of “Guanxi” pomelo and “Shatian” pomelo (Fig. 6). The isoflavone 7-O-beta-D-glucoside (19.3-fold), hesperetin 7-O-glucoside (3.9-fold), and leucopelargonidin (1.5-fold) were only accumulated in CLas-infected fruit pith of “Shatian” pomelo (Fig. 6; Table S9). By contrast, the hesperetin 7-O-glucoside (−5.3-fold), naringenin 7-O-beta-D-glucoside (−1.8-fold), poncirin (−2.1-fold), and hesperetin (−2.1-fold) were only reduced in CLas-infected fruit pith of “Shatangju” mandarin (Fig. 6; Table S9).

Comparative metabolites analysis identified a total of 18 altered terpenoids between CLas-infected and healthy fruit pith of three cultivars (Fig. 6). More accumulated terpenoids were identified in CLas-infected fruit pith of “Guanxi” pomelo and “Shatian” pomelo as compared to “Shatangju” mandarin (Fig. 6). A total of 12 and 9 terpenoids were accumulated in CLas-infected fruit pith of “Guanxi” pomelo and “Shatian” pomelo, respectively, while only 3 terpenoids accumulated in CLas-infected fruit pith of “Shatangju” mandarin (Fig. 6). Particularly, the terpenoids, secologanin, obacunone, and parthenin were only accumulated in CLas-infected fruit pith of “Guanxi” pomelo and “Shatian” pomelo and the gardenoside was increased in CLas-infected fruit pith all three cultivars (Fig. 6). The accumulation of seven terpenoids (lamioside, limonin, capsidiol, xanthoxic acid, harpagoside, menthone, and caryophyllene alpha-oxide) was only observed in CLas-infected fruit pith of “Guanxi” pomelo and four terpenoids (nomilin, bilobalide A, polygodial, and p-Cymene) were uniquely accumulated in CLas-infected fruit pith of “Shatian” pomelo (Fig. 6). By contrast, the levels of limonin (−2.5-fold), capsidiol (−1.4-fold), and perillyl alcohol (−1.9-fold) were lower in CLas-infected fruit pith of “Shatangju” mandarin than healthy fruit pith (Fig. 6; Table S10).

## DISCUSSION

The citrus fruit pith was able to support the multiplication of the CLas population to a high level, which made it an appropriate host tissue for CLas biology research. In this study, we first present the transcriptome landscape of CLas in the fruit pith of three cultivars with different tolerance to CLas, aiming to elucidate the biological function and pathways which involved in the adaption and proliferation of CLas within citrus phloem. Analysis of highly expressed CLas genes in the fruit pith of three cultivars showed that the majority of these genes belong to the pathways that were important for basal cellular function (Fig. 1A). These genes reflected the functions important for CLas colonization, growth, and multiplication in fruit pith. Among these genes, the outer membrane beta-barrel protein *OmpL* (CD16_RS03055) was highly expressed with TPM >3,000 in the fruit pith of three cultivars, particularly significantly up-regulated in the fruit pith of “Shatangju” mandarin as compared to others two pomelo cultivars (Table S5). The outer membrane beta-barrel proteins performed a variety of essential functions in cargo transport and signaling and were also vital for membrane biogenesis (32). The high expression of *OmpL* was also previously observed in the citrus leaf sample instead of psyllid (12), which indicated that it could play an important role in CLas survival and growth in citrus hosts. CD16_RS02370 (*flp1*), encoding the Flp family type IVb pilin, was highly expressed with a TPM value over 5,000 in the fruit pith of three cultivars (Table S5). The bacterial Type IV pilus was dynamic adhesive filaments on the surface of bacteria and showed a variety of functions in bacterial adherence, DNA uptake, twitching motility, bacterial interactions, and substrate transport (33). Interestingly, previous studies found that the CLas *flp1* exhibited significantly higher expression in citrus than in psyllid (12). The unique high expression of *flp1* in citrus host could facilitate the colonization of CLas in fruit pith. In addition, CD16_RS02445, encoding ferritin, was also highly expressed in the fruit pith of three cultivars and showed significantly higher expression levels in fruit pith of “Shatangju” mandarin than in “Shatian” pomelo (Table S5). In plant-pathogen interaction, plants used iron-withholding strategies as a defense mechanism to reduce pathogen virulence or to locally increase iron levels to activate a highly toxic oxidative burst (34). Bacteria can synthesize ferritin-like proteins to remove excess ferrous ions from the cytoplasm, which minimizes cell damage caused by iron toxicity (35). It is therefore that the induction of CLas ferritin could counteract or alleviate iron-regulated host immune responses and contribute to the colonization and growth of CLas in fruit pith.

CLas contained an intact TCA cycle apparatus that could play an important role in CLas growth in fruit pith. Our result found that CLas genes encoded enzymes involved in the TCA cycle were primarily induced in fruit pith with higher CLas concentration (“Shatangju” mandarin) as compared to the fruit pith with lower CLas concentration (“Guanxi” pomelo and “Shatian” pomelo) (Table 1; Table S3). CLas was thought to use exogenous fumarate, malate, succinate, and aspartate as carbon substrates for the TCA cycle (36, 37). The upregulation of most TCA-related enzymes, especially the isocitrate dehydrogenase, malate dehydrogenase, and fumarate hydratase (Table S3), suggested that the active CLas TCA cycle could contribute to the growth of CLas in fruit pith of “Shatangju” mandarin. Particularly, the fumaric acid and malic acid were both depleted in CLas-infected fruit pith of “Shatangju” mandarin (Table S8), indicating the utilization of two organic acids by CLas for feeding its TCA cycle. However, the isocitric acid, another intermediate of the TCA cycle, showed 190.5-fold higher in CLas-infected fruit pith of “Shatangju” mandarin but 2.0-fold lower in CLas-infected fruit pith of “Shatian” pomelo as compared to the healthy fruit pith (Fig. 6; Table S8). Previous studies argued that the noncyclic or shunted parts of the TCA pathway were more likely to function in CLas, consistent with the abundant availability of some TCA cycle intermediates in its extracellular milieu (38, 39). Indeed, some bacteria, such as *Escherichia coli*, operated the noncyclic variations in the TCA cycle to primarily generate biosynthetic precursors for lipids and amino acids under anaerobic or microaerophilic conditions (40). Based on the upregulation of some CLas TCA cycle-related enzymes and the metabolite changes of several TCA-associated compounds in CLas-abundant fruit pith, CLas might operate the noncyclic TCA pathways and more like to utilize malic acid and fumaric acid for its growth in fruit pith.

The CLas secretory effector has been found to play critical roles in CLas pathogenesis, manipulating plant immune responses and promoting CLas colonization (41–44). Several genes with the function of pathogenic effector or virulence factor were highly expressed in the fruit pith of three cultivars, particularly four (CD16_RS02255, 04385, 05110, and 05720) were up-regulated in fruit pith containing higher CLas concentration (“Shatangju” mandarin) (Table S3). Among these CLas effectors, the CD16_RS05720 was homologous to CLIBASIA_04250 of CLas strain Psy62, a recently identified effector that caused host phloem necrosis by disturbing the host normal pre-mRNA (45). CD16_RS05110 (homologous to CLIBASIA_05150) was found to target the Golgi apparatus and interact with critical host proteins involved in HLB development, disease resistance, and suppression of programmed cell death (46). CD16_RS02255 (homologous to CLIBASIA_03230) and CD16_RS04385 (homologous to CLIBASIA_04410) had been experimentally validated to contain signal peptide (47), although their functions had not been characterized yet. In addition, CD16_RS05225 (homologous to CLIBASIA_05315 or SDE1) was highly expressed in fruit pith of three cultivars and had been shown to exhibit various functions, such as inducing cell death and callose deposition in plant cells (48), inhibiting immune-related proteases activity by interaction with papain-like cysteine proteases (49), repressing the citrus DEAD-box RNA helicase and causing chlorosis symptoms (50). From a number of studies, CLas could secrete effector proteins to suppress or disturb plant immune responses and promote bacterial infection/multiplication (42, 43). The high expression induction of CLas effectors might contribute to CLas multiplication and disease development in fruit pith by suppressing the host immunity, particularly in “Shatangju” mandarin.

CLas phage or prophage of CLas had previously been found to play important roles in the pathogenicity, adaptability, and survival of the pathogen (13, 51–54). Three types of CLas-associated phage with large genomes (Type 1: SC1, Type 2: SC2, and Type 3: P-JXGC-3) (>30 kb) have been identified and characterized (3, 55). Type 1 phage encoded suspected lytic cycle genes and was found in lytic forms *in planta*, while Type 2 phage lacked lytic cycle genes and was involved in lysogenic conversion or replicating as a prophage excision plasmid (55). The recent study has found that the possible activation of the Type 1 phage lytic cycle could limit the proliferation of CLas strain at the early infection stage and led to the delayed infection of CLas in periwinkle (13). In this study, several Type 2 phage-specific genes involved in lysogenic conversion (protease, peroxidase, integrase, and exonuclease) were highly expressed in the fruit pith of all three cultivars (Table S5), which indicated that Type 2 phage might reside with CLas genome as a prophage form. The nearly single copy of Type 2 phage per CLas cell identified in CLas samples from fruit pith of “Shatian” pomelo and “Shatangju” mandarin agreed with the prophage form of Type 2 phage in CLas genome (Table 1). However, the Type 2 phage could replicate as a prophage excision plasmid in the fruit pith of “Guanxi” pomelo, since the multiple copies of Type 2 phage per CLas cell were observed in “Guanxi” pomelo (Table 1).

CLas contained a complete CRISPR/*cas* system in its prophage region (homologous region among three phages) (31). The previous study suggested that a pre-established CLas prophage could use its CRISPR/*cas* system to defeat the invasion of the other types of phage, which agreed with the observation of the predominance of single prophage type in the CLas population from southern China (31). In this study, CLas genes involved in the CRISPR/*cas* system significantly up-regulated or highly expressed in the fruit pith of “Shatian” pomelo as compared to other cultivars (Table S4). This indicated that the CRISPR/*cas* system might be active against the invasion of other phages in the fruit pith of “Shatian” pomelo. This was consistent with our observation that both Type 1 and Type 3 phage were in a very low abundance (0.1 copy per CLas cell) in CLas samples from fruit pith of “Shatian” pomelo (Table 1), which contain a pre-established Type 2 prophage in CLas genome with active CRISPR/*cas* system. In other words, the defeat of other phage, particularly the lytic Type 1 phage, by the pre-established Type 2 prophage with active CRISPR/*cas* system could help CLas to prevent the phage-induced bacterial lysis and indirectly contribute to the CLas growth in fruit pith. In addition, the phage/prophage encoded guanylate kinase and DNA polymerase were also highly expressed in fruit pith tissue (Table S4). The guanylate kinase was known to catalyze the ATP-dependent phosphorylation of GMP into GDP, which was then used as a DNA polymerase substrate for DNA replication (56). In consideration of their essential function in DNA replication, the high induction of prophage-encoded guanylate kinase and DNA polymerase could contribute to the DNA replication of CLas in fruit pith. Thus, the integration of the Type 2 phage genome as prophage in the CLas genome and the activation of prophage-encoded genes involved in the CRISPR/*cas* system and DNA synthesis could not only help CLas against the invasion of other lytic phages but also facilitate the chromosomal replication of CLas in fruit pith.

Amino acids in citrus phloem plants played a critical role in plant defense and their concentration was thought to be positively correlated to the degree of tolerance against HLB among citrus cultivars (19). The higher amounts of accumulated amino acids identified in CLas-infected fruit pith of two pomelo cultivars as compared to “Shatangju” mandarin suggested that they could play an important role in tolerance of two pomelo cultivars against CLas infection. Particularly, the phenylalanine (D-Phenylalanine and N-Acetyl-L-phenylalanine) and lysine (L-Lysine), which were implicated in plant defense (19, 27), were only accumulated in CLas-infected fruit pith of “Guanxi” pomelo and “Shatian” pomelo (Fig. 6). It was also found that the non-essential amino acid L-aspartic acids, which were positively correlated with citrus tolerance to CLas (20), was also accumulated in CLas-infected fruit pith of “Shatian” pomelo but decreased in the CLas-infected fruit pith of “Shatangju” mandarin (Fig. 6). In addition to the amino acid that related to plant defense against pathogen, the citrus phloem also contained the essential amino acids that were important for CLas survival, especially those amino acids that CLas was unable to make by itself. Analysis of genes involved in the amino acids biosynthetic pathways has found that CLas was unable to produce several amino acids, including histidine, tyrosine, thiamine, phenylalanine, tryptophan, asparagine, isoleucine, methionine, alanine, valine, leucine, and proline (36, 37). Interestingly, compared to the CLas genome, the presence of genes involved in the biosynthesis of essential amino acids phenylalanine and tyrosine in the genome of *Liberibacter crescens* was suggested to be in part of the culturable nature of *Liberibacter* crescens (57). We found that the L-Phenylalanine (9.3-fold) and L-Tryptophan (5.6-fold) only accumulated in CLas-infected fruit pith of “Shatangju” mandarin (with higher CLas concentration) as compared to “Guanxi” pomelo and “Shatian” pomelo (with lower CLas concentration) (Fig. 6; Table S6). CLas possessed a set of general L-amino acid ATP-binding cassette (ABC) transporters that were able to transport a variety of L-amino acids into the cell (37, 57). It is therefore interesting to hypothesize that CLas could use L-type of phenylalanine and tryptophan for its growth in fruit pith and the accumulation of L-Phenylalanine and L-Tryptophan in fruit pith of “Shatangju” mandarin could be beneficial for CLas growth and multiplication. However, the increased level of D-type of the phenylalanine in the fruit pith of two pomelo cultivars could be specifically related to their tolerance to CLas infection. Therefore, as one of the major altered compounds in CLas-infected fruit pith, some amino acids may play an important role in plant defense during CLas infection, while some could act as key nutrients supply for CLas survival in the host phloem.

Genome analysis found that the CLas was able to metabolize glucose, fructose, and xylulose but not mannose, galactose, rhamnose, or cellulose (36). Among these sugars, CLas might preferentially utilize fructose and lead to the impairment of sucrose loading and accumulation of sucrose and glucose, which lead to photosynthesis inhibition and starch accumulation in infected leaves (58). This was consistent with our observation that the fructose 1,6-bisphosphate was depleted and D-glucose was uniquely accumulated in CLas-infected fruit pith of “Shatangju” mandarin containing a higher CLas concentration (Fig. 6). The severe phloem blockage caused by CLas infection might hinder the transport of sugars to the juice sacs, resulting in the accumulation of sugars in the fruit pith, which benefited CLas growth. In addition, the maltotriose was highly accumulated in CLas-infected fruit pith of “Shatangju” mandarin (126.1-fold) but not in “Guanxi” pomelo and “Shatian” pomelo (Fig. 6; Table S7). Maltotriose was recognized as the indicator of the starch debranching process and was dramatically augmented in CLas-infected root (59, 60). The dramatic increase in maltotriose implied that the active breakdown of starch also occurred in CLas-infected fruit pith of “Shatangju” mandarin, which, in turn, provided the abundance of glucose for CLas growth and multiplication.

CLas lacked the genes involved in pathways to synthesize organic acids and must acquire organic acids from hosts (36, 37). Most of the organic acids identified in the citrus phloem sap of different cultivars were thought to be negatively correlated with citrus tolerance to CLas (20). CLas infection caused the reduction of organic acid content of phloem saps from the stem tissue of sweet orange “Valencia” (*Citrus sinensis*) and Madagascar periwinkle (*Catharanthus roseus*) (17). However, the phloem blockage caused by CLas infection was thought to be responsible for the localized organic acids availability and might benefit the CLas growth in localized phloem cells, leading to the uneven distribution of CLas within infected plants (17). In the fruits of most citrus species, organic acid content rapidly raised as the fruit expands and peaked at approximately 100–120 d after anthesis, after which it gradually declined (61). However, the phloem collapse caused by CLas infection may impair the transport of organic acids or influence the transformation and catabolism of organic acids in citrus fruit, which further causes the accumulation of organic acids in the phloem of fruit pith. Compared to “Shatian” pomelo, the higher accumulation of organic acids in CLas-infected fruit pith of “Guanxi” pomelo and “Shatangju” mandarin should be more favorable for CLas growth and proliferation, which was consistent with the higher CLas concentration in fruit pith of “Shatangju” mandarin and “Guanxi” pomelo than that observed in “Shatian” pomelo (Table 1).

As one of the largest classes of secondary metabolites produced in plants, flavonoids are known for their antimicrobial properties by inhibiting the growth of pathogens and enhancing plant defense during pathogen infection (62). CLas infection caused the accumulation of flavonoids in fruit pith tissue, particularly in “Guanxi” pomelo and “Shatian” pomelo (Fig. 6). Among accumulated flavonoids, the cyanidin 3-rutinoside, which exhibited >25-fold increase in CLas-infected fruit pith of “Guanxi” pomelo and “Shatian” pomelo, was reported to have antimicrobial activity against *Staphylococcus aureus* and *Escherichia coli* (63). Hesperidin has been reported to increase in orange leaf during blight-induced zinc deficiency (64), which suggested its participation in the plant response-to-stress mechanism. The antimicrobial properties of flavonoids had also been documented in isoflavone (65), pinocembrin (66), hesperetin (67), naringenin, and naringin (68), which were highly induced in fruit pith of “Guanxi” pomelo and “Shatian” pomelo (Fig. 6). The unique or stronger accumulation of flavonoids with antimicrobial activity could hinder the growth of CLas in fruit pith of “Guanxi” pomelo and “Shatian” pomelo. By contrast, the contents of hesperetin 7-O-glucoside, naringenin 7-O-beta-D-glucoside, and hesperetin were all decreased in CLas-infected fruit pith of “Shatangju” mandarin (Fig. 6), indicating more favorable microhabitats for CLas growth in fruit pith of “Shatangju” mandarin.

Terpenoids were the main volatile compounds identified in citrus plants and exhibited wide-ranging toxins involved in plant defense against various bacteria, fungi, and animals (69). Studies have been found that the citrus volatile terpenes known for their antimicrobial activity were in higher level in HLB-tolerant and moderately tolerant citrus cultivars compared to susceptible cultivars, indicating their critical roles involved in tolerance against HLB (27). Gardenoside, traditional Chinese medicine with antibacterial activity (70), was accumulated in CLas-infected fruit pith of all cultivars (Fig. 6). The limonin, exhibiting an effective antibacterial effect (71), was accumulated in CLas-infected fruit pith of “Guanxi” pomelo but reduced in “Shatangju” mandarin (Fig. 6). Previous study had found that the seed extract of “Shatian” pomelo, mainly contained naringin, deacetylnomilin, limonin, nomilin, and obacunone, exhibited antibacterial effects against *Bacillus subtilis*, *E. coli,* and *Xanthomonas citri* subsp. *citri* (72). It should be noted that the obacunone was accumulated in both CLas-infected “Guanxi” pomelo and “Shatian” pomelo and nomilin was uniquely accumulated in “Shatian” pomelo (Fig. 6). Compared to CLas-infected fruit pith of “Shatangju” mandarin, the higher number of accumulated terpenoids with antimicrobial properties in CLas-infected fruit pith of “Guanxi” pomelo and “Shatian” pomelo suggested they could increase the tolerance to CLas.

### Conclusion

In this study, we provided the genome-wide CLas gene expression profiles in fruit pith tissue of three citrus cultivars with different tolerance to CLas. CLas genes with basal cellular function were highly expressed in fruit pith. The induction of the CLas noncyclic TCA pathway and pathogenic effectors could promote the colonization and growth of CLas in fruit pith. We also found that the pre-established Type 2 prophage in the CLas genome, as well as the activity of its CRISPR/*cas* system, might enhance the phage resistance of CLas and indirectly facilitate CLas population growth in fruit pith. Metabolic profiling showed the accumulation of most sugars and organic acids in CLas-infected fruit pith, which could promote CLas growth. However, the accumulation of some amino acids, as well as the antimicrobial flavonoids and terpenoids, in CLas-infected fruit pith could be involved in tolerance to CLas and limit the growth of CLas. The finding of this study provides new insights into CLas multiplication in fruit pith and expands our understand of effect of metabolite changes on CLas growth.

## MATERIALS AND METHODS

### Plant materials

HLB-affected and healthy fruits of three cultivars were collected at fruit maturity season. These cultivars included “Shatian” pomelo (*Citrus maxima* cv. “Shatian Yu,” 6 years old), “Guanxi” pomelo (*C. maxima* cv. “Guanxi Yu,” ~6 years old), and “Shatangju” mandarin (*C. reticulata* Blanco cv. “Shatangju,” 6 years old) (Fig. S2). The HLB-affected and healthy fruits of “Shatian” pomelo and “Guanxi” pomelo were originally from Dabu country (24°22′23″N, 116°41′12″E) of Meizhou city in Guangdong province and collected in October 2022. The HLB-affected and healthy fruits of “Shatangju” mandarin were originally collected from Boluo country of Huizhou city (23°07′94″N, 114°41′26″E) in Guangdong province in December 2022. For each cultivar, five HLB-affected trees and five healthy trees were selected. A total of five fruits were collected from each healthy or HLB-affected tree. For each fruit, five to eight piths were pulled out as one sample and then immediately put into the liquid nitrogen before being taken to the laboratory for DNA and RNA extraction.

### DNA and RNA extraction

For DNA extraction, 100 mg of fruit pith tissue was collected and initially ground with an MP FastPrep −24 Grinder (MP Biomedicals LLC, Santa Ana, CA, United States) at a speed of 4 M/S for 20 s. Total DNA was extracted using an E.Z.N.A. HP Plant DNA Kit (OMEGA Bio-Tek Co., Guangdong, China) according to the manufacturer’s manual. For RNA extraction, 100 mg of frozen fruit pith was ground in liquid nitrogen. The E. Z. N. A. Plant RNA Kit (OMEGA Bio-Tek Co., Guangdong, China) was used for RNA extraction from fruit pith tissue. The concentration of all extracted DNA and RNA samples was determined using Qubit 2.0 (Thermo Fisher Scientific Inc., Waltham, MA, United States). The quality of RNA samples was examined by Agilent 2100 (Agilent Technologies Inc., Santa Clara, CA, U.S.A.) before sequencing.

### Quantification of CLas and the associated phage

Quantification analysis of CLas in fruit pith was referred to a previously developed SYBR real-time PCR procedure with specific primer CLas-4G/HLBr (73). Each PCR mixture (20 µL) contained 10 µL of Bestar qPCR Master Mix (DBI Bioscience, Shanghai, China), 0.5 µL of each forward and reverse primer (10 µM), 1 µL of DNA template (∼25 ng), and 8 µL of ddH_2_O. All SYBR Green reactions were performed on a Bio-Rad Real-Time PCR apparatus (Bio-Rad Laboratories Inc., Hercules, CA, U.S.A.) under the following procedure: 95°C for 3 min, followed by 40 cycles at 95°C for 10 s, 60°C for 30 s, and 72°C for 30 s, with fluorescence signal capture at the end of each 60°C step. PCR result (Ct value) was obtained using Bio-Rad CFX Manager 2.1 software with automated baseline settings and threshold. DNA sample with a Ct value less than 32 was considered CLas positive. The standardized equation (y = −3.310 x +37.463, R^2^ = 0.995) was developed for CLas quantification. Briefly, a recombinant plasmid contained the CLas-4G/HLBr region was used for the construction of the standard equation. The concentration of recombinant plasmid and all DNA extracts was determined by using Qubit 2.0 (Thermo Fisher Scientific Inc.). The copy number of plasmid was calculated according to the following formula: the number of copies = (amount in nanograms × Avogadro number)/(length in base pairs × 1 × 10^9^ ×650). Quantification of CLas for each sample was indicated as CLas cells per nanograms of total DNA.

Quantification of phage in CLas samples was determined by the threshold cycle methods with a CLas-specific primer set (CLas4G/HLBr) and three phage type-specific primer sets (Type 1, SC1-045F/SC1-045R; Type 2, SC2-040F/SC2-040R; and Type 3, P-JXGC_08F/P-JXGC_08R) (74). The density of phage in “*Ca*. Liberibacter asiaticus” cells was indicated as copy number of phage per CLas cell with ΔCt method according to a previous study (75), that is, R = 2^−ΔCt^, ΔCt = Ct (SC1-045F/SC1-045R, SC2-040F/SC2-040R, or P-JXGC_08F/P-JXGC_08R)−Ct (primer set target single copy gene of CLas). The Ct value of primer set target single copy gene of CLas was converted from the Ct value generated by primer set CLas4G/HLBr (targeted to three copies of the CLas 16S rRNA gene) with the equation: Ct (CLas4G/HLBr) + X, where X is 1.585.

### RNA sequencing and CLas transcriptomic analysis

Three biological replicates of RNA samples extracted from fruit pith of HLB-affected fruits were selected for dual-RNA Seq analysis. Library preparation for dual RNA-Seq was performed with a TruSeq RNA library Prep Kit (Illumina, San Diego, CA, United States) by removing rRNA from total plant RNA. High-throughput sequencing was carried out on an Illumina XTen platform with 150 bp paired-end reads provided by the Novogene Company (Novogene, Co., Ltd., Beijing, China). Dual RNA-Seq data of CLas-infected fruit pith contained citrus host reads and CLas reads. In this study, only CLas RNA-Seq data were used to reveal the CLas transcriptome landscape. Thus, the clean data of RNA samples extracted from CLas-infected fruit pith were mapped to CLas strain A4 genome (CP010804.2) and three phage sequences, including Type 1 (represented by P-YNBC-1), Type 2 (represented by P-A4-2), and Type 3 (represented by P-JXGC-3, KY661963.1) by CLC Genomic Workbench v20 (Qiagen Bioinformatics, Aarhus, Denmark). P-YNBC-1 was the Type 1 prophage region (from nucleotide position 1,187,948 to 1,230,892) of CLas strain YNBC (CP118771.1) and P-A4-2 was the Type 2 prophage region (from nucleotide position 1,189,877 to 1,603) of CLas strain A4 (CP010804.2). The table of “Total Gene Reads” was generated by summarizing of reads mapped to each CLas gene. The transcripts per kilobase million (TPM) method was used for the normalization of each RNA-seq, that is, TPM = A × 10^6^ ×1/∑(A), where A = total reads mapped to gene ×10^3^/gene length in bp. RNA-Seq data from the fruit pith of “Shatangju” mandarin was selected as the control to identify the differential expression genes (DEGs) of CLas in the fruit pith of “Shatian” pomelo and “Guanxi” pomelo. DEGs between CLas samples from different cultivars were identified through CLC Genomic Workbench v20 based on the multi-factorial statistics Generalized Linear Model (GLM) with a cutoff setting as Log2 fold change ≥ | 1 | and *P*-value < 0.05. Functional annotation and ortholog assignment of all identified DEGs were further analyzed using eggNOG mapper (76). Heat maps of DEGs were generated in TBtools software (77). To validate the results of DEGs identified by RNA sequencing, 10 CLas genes, including ribonucleotide-diphosphate reductase subunit beta, DEAD/DEAH box helicase, NADP-dependent isocitrate dehydrogenase, malate dehydrogenase, outer membrane beta-barrel protein, ferritin, and secretion effector genes, were selected for reverse transcription-qPCR (RT-qPCR) (Table S11). The 16S rRNA genes of CLas were used as an internal reference with primer set CLas4G/HLBr (73). The fold change of each gene from the RT-qPCR result was calculated by the comparative CT method (2^–∆∆CT^). The log2 fold change was generated from the ratio of the relative expression value of CLas strain from “Shatian” pomelo or “Guanxi” pomelo to that of “Shatangju” mandarin. For each gene, log2 fold change values of RT-qPCR were compared with those of the same gene from RNA-Seq analyses. The expression profiles of 10 selected CLas genes generated by RT-qPCR were consistent with RNA-Seq data (*R^2^* = 0.95) (Fig. S3), indicating the reliability of RNA-Seq analysis in this study.

### Untargeted metabolomic analysis and data processing

For each cultivar, three biological replicates from CLas-infected and healthy fruit pith were collected for metabolomic analysis. Metabolite extraction for each sample was performed according to a previous study (78). Each fruit pith tissue sample (50 mg) was added with 600 µL LC-MS grade 85% MeOH (Fisher Scientific, Loughborough, UK) containing 2-Amino-3-(2-chloro-phenyl)-propionic acid (4 ppm, internal standard) (Aladdin, Shanghai, China) and then ground by a tissue grinder under into 60 Hz for 90 s. The ground samples were then ultrasound-treated for 15 min and centrifuged at 14,000 rpm for 15 min at 4°C. After centrifugation, the supernatant was separated by filtrating through a 0.22-µm membrane, and the filtrate was collected and added into the detection bottle for liquid chromatography-mass spectrometry (LC-MS) analyses. The LC-MS analyses were performed by Vanquish UHPLC System (Thermo Fisher Scientific, USA) by Suzhou PANOMIX Biomedical Tech Co., LTD (Jiangsu, China). Chromatography was carried out with an Acquality UPLC HSS T3 column (150 × 2.1 mm, 1.8 µm) (Waters, Milford, MA, USA), operated at 40°C with the flow rate and injection volume setting at 0.25 mL/min and 2 µL, respectively. For LC-ESI (-)-MS analysis, the analytes were carried out with (A) acetonitrile and (B) ammonium formate (5 mM). For LC-ESI (+)-MS analysis, the mobile phases consisted of (C) 0.1% formic acid in acetonitrile (vol/vol) and (D) 0.1% formic acid in water (vol/vol). The separation of compounds was conducted under the following gradient: 0–1 min, 2% A/C; 1–9 min, 2%–50% A/C; 9–12 min, 50%–98% A/C; 12–13.5 min, 98% A/C; 13.5–14 min, 98%–2% A/C; 14– 20 min, 2% A/C. Mass spectrometric detection of metabolites was performed on Orbitrap Exploris 120 (Thermo Fisher Scientific, USA) with an ESI ion source. Simultaneous MS1 and MS/MS (Full MS-ddMS2 mode, data-dependent MS/MS) acquisition was applied with MS1 resolving power setting as 60000 FWHM and MS/MS resolving power setting as 15000 FWHM.

The raw mass spectrometer off-camera file was normalized into mzXML file format using MSConvert tool from the Proteowizard software package (v3.0.8789) (79) and processed using the XCMS for feature detection, retention time correction, and alignment (80). The parameter settings were as follows: bw = 2, ppm = 15, peak width = c[5,30], mzwid = 0.015, mzdiff = 0.01, and method = “centWave.” Metabolite identification was first confirmed based on the precise molecular weight, followed by MS/MS fragmentation mode for HMDB (http://www.hmdb.ca), massbank (http://www.massbank.jp/), LipidMaps (http://www.lipidmaps.org), mzcloud (https://www.mzcloud.org), KEGG (http://www.genome.jp/kegg/), and self-built substance library with parameter setting as ppm <30. The robust LOESS signal correction (QC-RLSC) was applied for data normalization to correct for any systematic bias (81). Only ion peak with relative standard deviation (RSD) less than 30% in the QC samples were kept to proper metabolite identification. The Ropls software was used for all multivariate data analyses and modeling based on principal component analysis (PCA), partial least squares-discriminant analysis (PLS-DA), and orthogonal partial least-square discriminant analysis (OPLS-DA) (82). The OPLS-DA was used to determine the discriminating metabolites using the variable projection on importance (VIP). The *P* value, VIP produced by OPLS-DA, fold change (FC) was applied to discover the contributable variable for classification. Metabolites with the |fold change| > 1.5 fold (*P*-value value <0.05 and VIP > 1) between CLas-infected and healthy control were considered statistically significant. The ggplot2 package (https://ggplot2.tidyverse.org/) of R software was used to draw the bubble charts for KEGG enrichment results.

## Data Availability

The RNA-seq data that support the findings of this study are available in the NCBI Short Read Archive under BioProject in the NCBI Short Read Archive under BioProject PRJNA854212. Metabolome analysis data that support the findings of this study have been deposited into CNGB Sequence Archive (CNSA) of China National GeneBank DataBase (CNGBdb) with accession number CNP0005371. The mirror plots of key identified metabolites in this study were included in Fig. S4.

## SUPPLEMENTAL MATERIAL

Fig. S1 to Fig. S4, Tables S1 to Table S11 spectrum.04052-23-s0001.pdf Supplemental figures and tables.

ASM does not own the copyrights to Supplemental Material that may be linked to, or accessed through, an article. The authors have granted ASM a non-exclusive, world-wide license to publish the Supplemental Material files. Please contact the corresponding author directly for reuse.
