# Supplementary material for: Integrated bacterial transcriptome and host metabolome analysis reveals insights into “Candidatus Liberibacter asiaticus” population dynamics in the fruit pith of three citrus cultivars with different tolerance
Source: Microbiol Spectr. 2024 Mar 5;12(4):e04052-23. doi: 10.1128/spectrum.04052-23 (PMC10986616; doi:10.1128/spectrum.04052-23)
Supplement: Fig. S1 to Fig. S4, Tables S1 to Table S11 — Supplemental figures and tables. [file spectrum.04052-23-s0001.pdf]

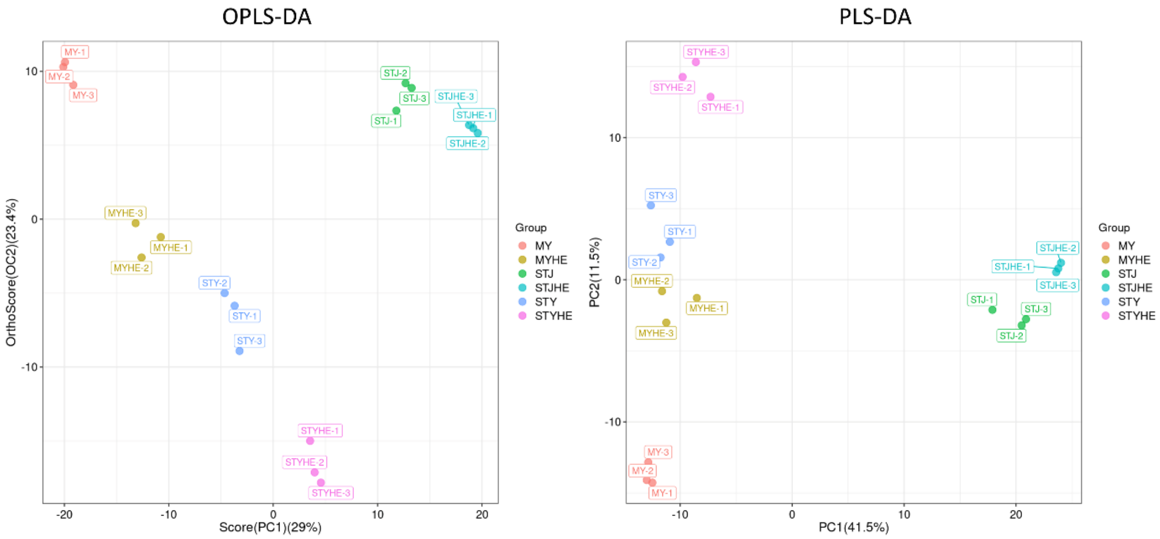

Figure S1. Partial least squares-discriminant analysis (PLS-DA) and orthogonal PLS-DA (OPLS-DA) score plots of total sample metabolites from "*Candidatus* Liberibacter asiaticus"-infected and healthy fruit pith of three cultivars. MY, CLas-infected 'Guanxi' pomelo. MYHe, Healthy 'Guanxi' pomelo. STY, CLas-infected 'Shatian' pomelo. STYHe, Healthy 'Shatian' pomelo. STJ, CLas-infected 'Shatangju' mandarin. STJHe, Healthy 'Shatangju' mandarin.

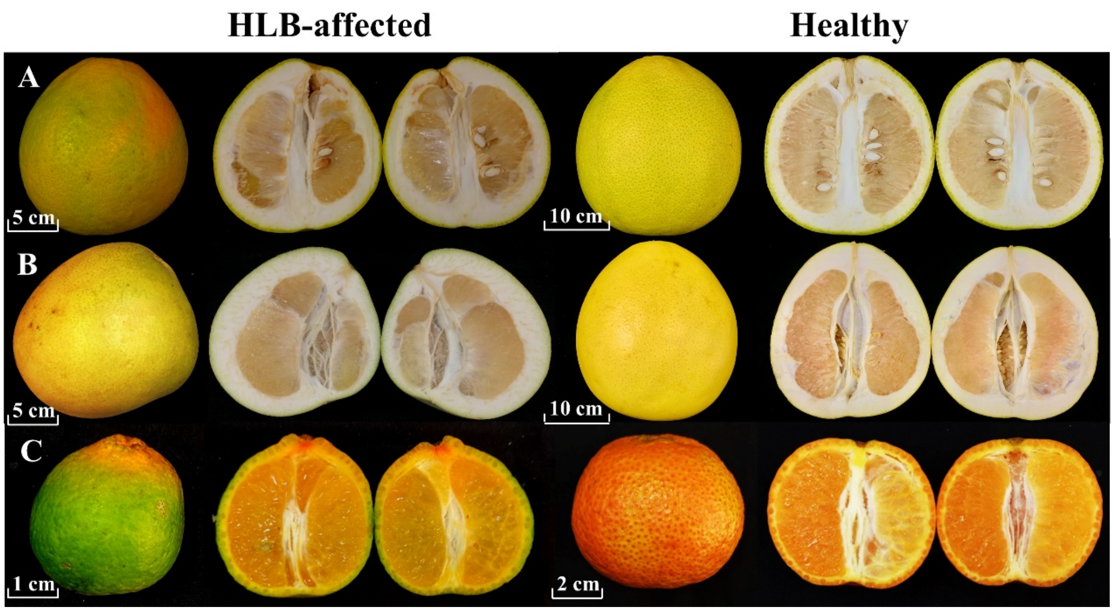

Figure S2. Huanglongbing-affected citrus fruit from three citrus cultivars and healthy citrus fruit used in this study. A. *Citrus maxima* cv. ‘Shatian Yu’. B. *C. maxima* cv. ‘Guanxi’ pomelo. C. *C. reticulata* Blanco cv. ‘Shatangju’.

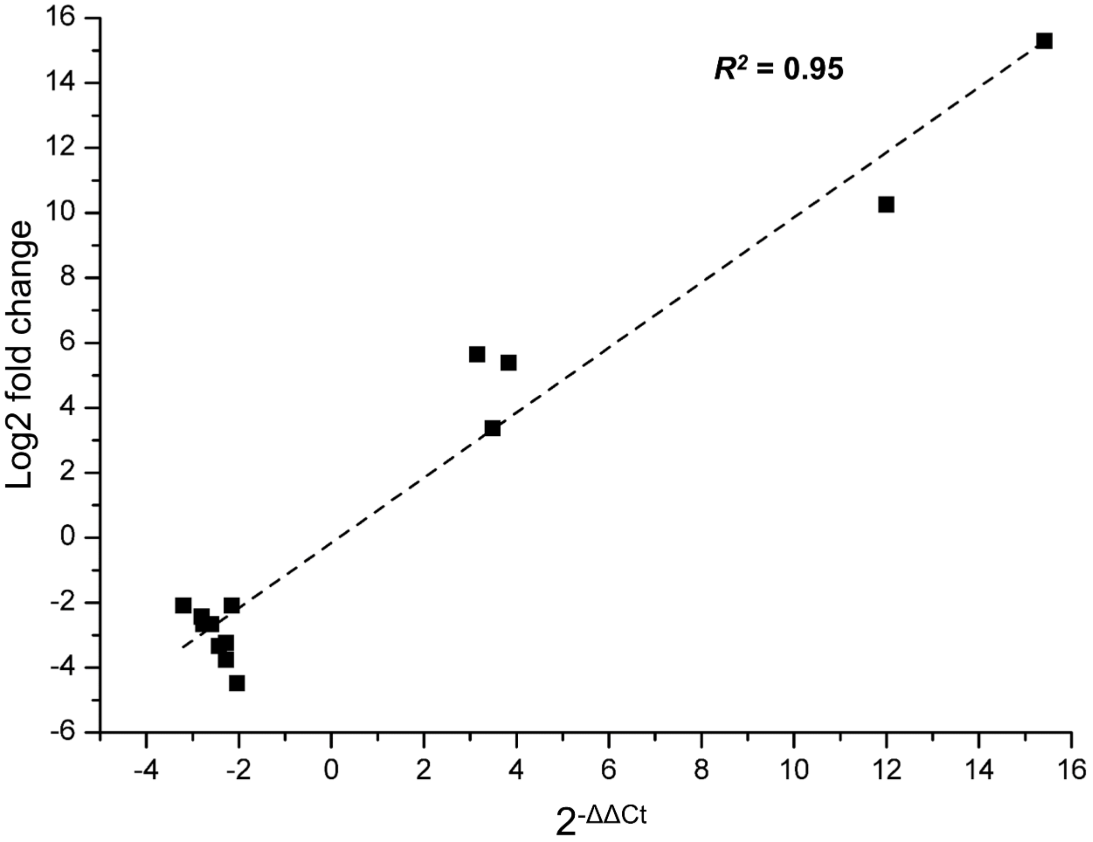

Figure S3. Correlation of ten "*Candidatus* Liberibacter asiaticus" gene expression (log2Fold Change) between RNA-seq and qPCR validation. $2^{-\Delta\Delta Ct}$ value was obtained from relative quantification of Real-time qPCR.

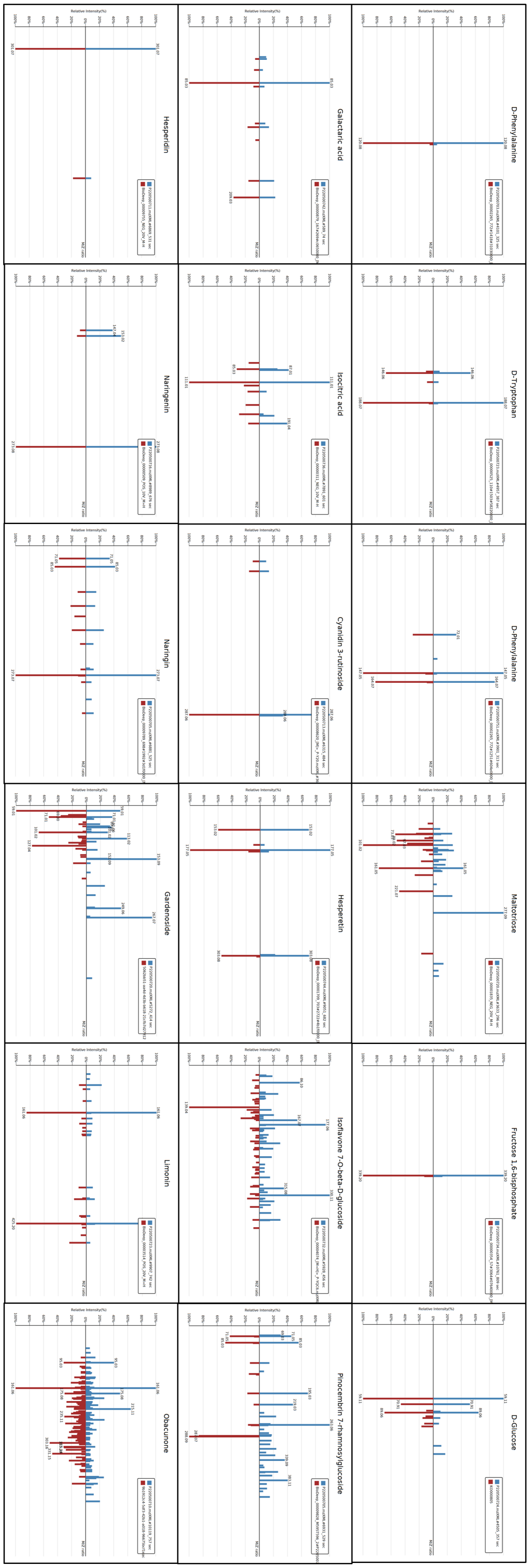

Figure S4. The mirror plots of key identified metabolites in this study.

Table S1. General information of RNA-Seq data generated from "*Candidatus* Liberibacter asiaticus" (CLas)-infected fruit pith of three cultivars.

Sample name*	Clean reads	Q20(%)	Q30(%)	Mapped to CLas genome (% of total reads) *
MY_1	144,180,112	96.54	90.76	435,389 (1.21%)
MY_2	138,023,328	96.83	91.33	445,024 (1.31%)
MY_3	137,888,298	96.44	90.56	464,401 (1.32%)
STY_1	143,782,340	96.53	90.78	377,773 (1.23%)
STY_2	143,038,498	95.69	89.4	683,562 (1.94%)
STY_3	132,140,706	96.57	90.95	368,902 (1.31%)
STJ_1	135,738,606	95.68	87.66	562,556 (1.29%)
STJ_2	141,224,238	95.59	87.28	562,926 (1.22%)
STJ_3	130,406,724	95.54	88.26	539,212 (1.30%)

*MY, CLas-infected 'Guanxi' pomelo; STY, CLas-infected 'Shatian' pomelo; STJ, CLas-infected 'Shatangju mandarin'.

Table S2. Pearson correlation of RNA-Seq data from "*Candidatus* Liberibacter asiaticus" (CLas)-infected fruit pith of three cultivars.

Sample ID	MY_1	MY_2	MY_3	STY_1	STY_2	STY_3	STJ_1	STJ_2	STJ_3
MY_1	1	0.992	0.992	0.936	0.904	0.937	0.82	0.821	0.817
MY_2	0.992	1	0.992	0.935	0.899	0.936	0.821	0.822	0.817
MY_3	0.992	0.992	1	0.937	0.903	0.938	0.822	0.823	0.818
STY_1	0.936	0.935	0.937	1	0.961	0.988	0.806	0.807	0.803
STY_2	0.904	0.899	0.903	0.961	1	0.961	0.766	0.768	0.764
STY_3	0.937	0.936	0.938	0.988	0.961	1	0.803	0.805	0.801
STJ_1	0.82	0.821	0.822	0.806	0.766	0.803	1	0.99	0.99
STJ_2	0.821	0.822	0.823	0.807	0.768	0.805	0.99	1	0.991
STJ_3	0.817	0.817	0.818	0.803	0.764	0.801	0.99	0.991	1

*MY, CLas-infected 'Guanxi' pomelo; STY, CLas-infected 'Shatian' pomelo; STJ, CLas-infected 'Shatangju mandarin'.

Table S3. Differentially expressed genes from chromosomal region of "*Candidatus* Liberibacter asiaticus".

Locus_tag	TPM			Log$_2$ fold change		Product
	'Guanxi' pomelo	'Shatian' pomelo	'Shatangju' mandarin	'Guanxi' pomelo	'Shatian' pomelo	
CD16_RS04295	1,020	1,163	83	3.4	3.6	ribonucleotide-diphosphate reductase subunit beta
CD16_RS05750	63	250	14	1.9	3.9	DEAD/DEAH box helicase
CD16_RS00025	732	653	177	1.8	1.7	hypothetical protein
CD16_RS03945	892	815	1,505	-1.0	-1.1	DUF1153 domain-containing protein
CD16_RS05530	160	103	273	-1.0	-1.6	hypothetical protein
CD16_RS00830	1,174	767	2,025	-1.0	-1.6	30S ribosomal protein S9
CD16_RS00245	354	282	622	-1.1	-1.4	hypothetical protein
CD16_RS04350	38	116	68	-1.1	0.5	hypothetical protein
CD16_RS04380	39	65	69	-1.1	-0.3	hypothetical protein
CD16_RS02920	3,337	5,004	6,012	-1.1	-0.5	Hsp20 family protein
CD16_RS01460	1,374	887	2,486	-1.1	-1.7	50S ribosomal protein L25/general stress protein Ctc
CD16_RS02255	2,082	1,597	3,788	-1.1	-1.5	hypothetical protein
CD16_RS01455	1,617	1,379	2,976	-1.1	-1.3	CarD family transcriptional regulator
CD16_RS00340	389	235	726	-1.1	-1.9	50S ribosomal protein L31
CD16_RS00895	2,571	1,489	4,982	-1.2	-2.0	lytic murein transglycosylase
CD16_RS03055	5,146	3,700	10,103	-1.2	-1.7	outer membrane beta-barrel protein
CD16_RS05720	324	505	664	-1.3	-0.6	hypothetical protein
CD16_RS04385	1,752	1,619	3,606	-1.3	-1.4	hypothetical protein
CD16_RS01570	742	670	1,604	-1.4	-1.5	transcription elongation factor GreA
CD16_RS00070	768	821	1,715	-1.4	-1.3	50S ribosomal protein L7/L12
CD16_RS05725	22	20	52	-1.5	-1.6	hypothetical protein
CD16_RS05690	90	172	234	-1.6	-0.7	hypothetical protein
CD16_RS03210	483	503	1,267	-1.6	-1.6	hypothetical protein
CD16_RS02890	913	718	2,541	-1.7	-2.0	chromosomal replication initiator protein DnaA
CD16_RS04035	1,449	1,248	4,059	-1.7	-1.9	cold shock domain-containing protein
CD16_RS04890	2,316	2,381	7,267	-1.9	-1.8	hypothetical protein
CD16_RS04880	2,343	2,797	7,849	-2.0	-1.7	hypothetical protein
CD16_RS02385	32	47	109	-2.0	-1.4	Flp family type IVb pilin
CD16_RS04885	1,651	2,305	6,146	-2.1	-1.6	hypothetical protein
CD16_RS04895	1,026	498	6,135	-2.8	-3.8	hypothetical protein
CD16_RS05580	374	3,684	489	-0.6	2.7	hypothetical protein
CD16_RS05550	830	1,959	593	0.2	1.5	intrrupted gp229, phage associated protein
CD16_RS05755	63	122	41	0.4	1.3	hypothetical protein
CD16_RS05740	51	58	23	0.9	1.1	hypothetical protein
CD16_RS01975	734	1,239	514	0.3	1.0	protoporphyrinogen oxidase HemJ
CD16_RS05715	50	86	36	0.2	1.0	hypothetical protein
CD16_RS02710	770	710	1,215	-0.9	-1.0	carbonic anhydrase

CD16_RS00985	731	391	677	-0.1	-1.0	hypothetical protein
CD16_RS02540	1,153	922	1,599	-0.7	-1.0	nucleotide exchange factor GrpE
CD16_RS01730	568	493	859	-0.8	-1.0	DNA gyrase inhibitor YacG
CD16_RS04910	673	498	868	-0.6	-1.0	ATPase
CD16_RS03985	1,052	747	1,302	-0.6	-1.0	NADP-dependent isocitrate dehydrogenase
CD16_RS05430	720	479	837	-0.5	-1.0	hypothetical protein
CD16_RS02320	796	738	1,296	-0.9	-1.0	signal peptide peptidase SppA
CD16_RS01015	1,238	818	1,454	-0.5	-1.1	30S ribosomal protein S18
CD16_RS00235	779	703	1,266	-0.9	-1.1	ETC complex I subunit
CD16_RS01735	1,178	747	1,361	-0.5	-1.1	translation initiation factor IF-1
CD16_RS05520	836	570	1,048	-0.6	-1.1	hypothetical protein
CD16_RS03555	1,651	1,159	2,151	-0.6	-1.1	superoxide dismutase
CD16_RS03940	578	351	653	-0.4	-1.1	hypothetical protein
CD16_RS02555	397	298	566	-0.8	-1.1	hypothetical protein
CD16_RS00310	56	47	90	-0.9	-1.2	ribonucleotide-diphosphate reductase subunit beta
CD16_RS00825	1,558	1,206	2,338	-0.8	-1.2	50S ribosomal protein L13
CD16_RS04710	978	719	1,403	-0.8	-1.2	malate dehydrogenase
CD16_RS01795	856	648	1,267	-0.8	-1.2	ribonuclease P protein component
CD16_RS05110	3,377	2,814	5,562	-1.0	-1.2	hypothetical protein
CD16_RS01595	765	596	1,202	-0.9	-1.2	isoleucine--tRNA ligase
CD16_RS01405	194	112	227	-0.5	-1.2	DUF1674 domain-containing protein
CD16_RS05745	360	181	368	-0.3	-1.2	hypothetical protein
CD16_RS00320	678	525	1,119	-1.0	-1.3	class II fumarate hydratase
CD16_RS04065	438	295	642	-0.8	-1.3	dTDP-4-dehydrorhamnose 3,5-epimerase
CD16_RS00510	286	202	449	-0.9	-1.4	YggT family protein
CD16_RS00165	642	431	959	-0.8	-1.4	hypothetical protein
CD16_RS04875	650	366	836	-0.6	-1.4	hypothetical protein
CD16_RS02485	1,153	760	1,779	-0.9	-1.4	30S ribosomal protein S16
CD16_RS01170	244	145	343	-0.7	-1.5	50S ribosomal protein L32
CD16_RS02445	3,777	2,577	6,200	-1.0	-1.5	ferritin
CD16_RS00900	114	61	151	-0.6	-1.5	hypothetical protein
CD16_RS05205	1,195	715	1,846	-0.9	-1.6	helix-turn-helix transcriptional regulator
CD16_RS02895	2,887	1,536	4,020	-0.7	-1.6	30S ribosomal protein S20
CD16_RS05915	2,184	777	2,340	-0.3	-1.8	hypothetical protein
CD16_RS05850	22	0	22	-0.2	-5.1	DnaB-like helicase N-terminal domain-containing protein

Table S4. Expression data of genes from phage/prophage region.

Phage Types	Gene locus	Gene description	TPM (Fold change)*		
			'Guanxi' pomelo	'Shatian' pomelo	'Shatangju' mandarin
Type 1 / Type 2 / Type 3	PP174_05330/PP174_05525/CD16_RS05605/B2I23_05585	DNA polymerase	746	1,310 (2.0)	566
Type 1 / Type 2 / Type 3	PP174_05335/PP174_05530/CD16_RS05610/B2I23_05590	VRR-NUC domain-containing protein	218	520	244
Type 1 / Type 2 / Type 3	PP174_05340/PP174_05535/CD16_RS05405/CD16_RS05615/B2I23_05410/B2I23_00005	DEAD/DEAH box helicase	987	1,138	673
Type 1 / Type 2 / Type 3	PP174_05345/PP174_05540/CD16_RS00005/CD16_RS05410/B2I23_00010/B2I23_05415	DNA ligase	273	285	240
Type 1 / Type 2 / Type 3	PP174_05350/CD16_RS05415/B2I23_05420	guanylate kinase	3,214	4,173	1,900
Type 1 / Type 2 / Type 3	PP174_05355/CD16_RS05420/B2I23_05425	hypothetical protein	731	769	1,015
Type 1 / Type 2 / Type 3	PP174_05445/CD16_RS05515/B2I23_05490	terminase	889	1,395	1,145
Type 1 / Type 2 / Type 3	PP174_05450/CD16_RS05520/B2I23_05495	transposase	836	570	1,048
Type 1 / Type 2 / Type 3	PP174_05465/CD16_RS05535/B2I23_05510	hypothetical protein	538	996	708
Type 1 / Type 2 / Type 3	PP174_05470/CD16_RS05540/B2I23_05515	hypothetical protein	167	303	165
Type 1 / Type 2 / Type 3	PP174_05475/CD16_RS05545/B2I23_05520	hypothetical protein	244	365	218
Type 1 / Type 2 / Type 3	PP174_05480/CD16_RS05550/B2I23_05525	hypothetical protein	830	1,959 (2.8)	593
Type 1 / Type 2 / Type 3	PP174_05485/CD16_RS05555/B2I23_05535	hypothetical protein	742 (-2.0)	1,157	1,231
Type 1 / Type 2 / Type 3	PP174_05490/CD16_RS05560/B2I23_05540	DNA primase	883	966	700
Type 1 / Type 2 / Type 3	PP174_05495/CD16_RS05565/B2I23_05545	hypothetical protein	288	329	319
Type 1 / Type 2 / Type 3	PP174_05500/CD16_RS05580/B2I23_05560	hypothetical protein	374	3,684 (6.4)	489
Type 1 / Type 2 / Type 3	PP174_05505/CD16_RS05585/B2I23_05565	hypothetical protein	1,726	2662 (2.0)	1,179
Type 1 / Type 2 / Type 3	PP174_05510/CD16_RS05590/B2I23_05570	DUF2800 domain-containing protein	635	1,082	747
Type 1 / Type 2 / Type 3	PP174_05515/CD16_RS05595/B2I23_05575/B2I23_05530	phage repressor protein	281	265	286

Type 1 / Type 2 / Type 3	PP174_05520/CD16_RS05600/B2I23_05580	DUF2815 family protein	396	724 (2.0)	315
Type 1 / Type 2	PP174_05360/CD16_RS05915	hypothetical protein	2,184	777 (-3.5)	2,340
Type 1 / Type 2 / Type 3	PP174_05365/CD16_RS05430/B2I23_05435	hypothetical protein	720	479 (-2.0)	837
Type 1 / Type 2 / Type 3	PP174_05370/CD16_RS05435/CD16_RS05450	tail protein	625	737	573
Type 1 / Type 2	PP174_05375/CD16_RS05455	putative phage-related protein	929	592	713
Type 1 / Type 2 / Type 3	PP174_05455/CD16_RS05525/B2I23_05500	helix-turn-helix domain-containing protein	952	1,537	827
Type 1 / Type 2 / Type 3	PP174_05460/CD16_RS05530/B2I23_05505	hypothetical protein	160 (-2.0)	103 (-3.1)	273
Type 1 / Type 2 / Type 3	PP174_00005/CD16_RS00010/B2I23_00015	guanylate kinase	1,012	880	585
Type 2 / Type 3	CD16_RS05395/B2I23_05400	DNA polymerase	2,853	2,581	1,787
Type 2 / Type 3	CD16_RS05400/B2I23_05405	VRR-NUC domain-containing protein	233	251	215
Type 2 / Type 3	CD16_RS05570/B2I23_05550	putative phage-related protein	302	592	432
Type 2 / Type 3	CD16_RS05575/B2I23_05555	hypothetical protein	212	395	199
Type 2 / Type 3	CD16_RS05750/B2I23_00005	DEAD/DEAH box helicase	63 (3.8)	250 (15.4)	14
Type 1	PP174_05380	Phage-related protein	NA	346	NA
Type 1	PP174_05385	Endolysin	NA	261	NA
Type 1	PP174_05390	head protein	NA	472	NA
Type 1	PP174_05395	Colicin IA; PRK02224 superfamily	NA	212	NA
Type 1	PP174_05400	cell envelope integrity protein TolA	NA	521	NA
Type 1	PP174_05405	Tail protein	NA	824	NA
Type 1	PP174_05410	Tail protein	NA	641	NA
Type 1	PP174_05415	phage capsid protein	NA	1,170	NA
Type 1	PP174_05420	hypothetical protein	NA	587	NA
Type 1	PP174_05425	putative phage-related glutathione peroxidase	NA	293	NA
Type 1	PP174_05430	Portal protein	NA	600	NA
Type 1	PP174_05435	Holin	NA	619	NA
Type 1	PP174_05440	hypothetical protein	NA	235	NA
Type 2	CD16_RS05435	putative phage-related protein; PRK08999 superfamily	917	511	732
Type 2	CD16_RS05440	putative phage-related [illegible]	2,664	2,252	2,540

Type 2	CD16_RS05445	putative phage-related protein	437	432	362
Type 2	CD16_RS05460	putative phage-related protein	1,871	1,363	1,375
Type 2	CD16_RS05465	head protein	1,544	1,028	1,125
Type 2	CD16_RS05470	putative phage-related protein	3,667	2,615	2,806
Type 2	CD16_RS05475	integrase	967	686	821
Type 2	CD16_RS05480	exonuclease	1,259	815	1,248
Type 2	CD16_RS05485	tail protein	2,663	1,657	2,012
Type 2	CD16_RS05490	hypothetical protein	605	537	448
Type 2	CD16_RS05495	hypothetical protein	758	519	647
Type 2	CD16_RS05500	protease	1,384	918	1,098
Type 2	CD16_RS05505	putative phage-related glutathione peroxidase	1,080	799	1,319
Type 2	CD16_RS05510	phage tail protein	3,030	2,120	2,187
Type 2	CD16_RS05575	hypothetical protein	212	395	199
Type 3	B2I23_05430	signal recognition particle receptor protein FtsY alpha subunit	NA	3,256	NA
Type 3	B2I23_05440	hypothetical protein	NA	86	NA
Type 3	B2I23_05445	hypothetical protein	NA	14	NA
Type 3	B2I23_05450	hypothetical protein	NA	205	NA
Type 3	B2I23_05455	hypothetical protein	NA	111	NA
Type 3	B2I23_05460	DNA helicase related protein	NA	92	NA
Type 3	B2I23_05465	DEAD/DEAH box helicase	NA	363	NA
Type 3	B2I23_05470	restriction endonuclease subunit S	NA	717	NA
Type 3	B2I23_05475	restriction endonuclease subunit S	NA	259	NA
Type 3	B2I23_05480	DNA methyltransferase	NA	518	NA
Type 3	B2I23_05485	DNA methylase	NA	424	NA

*NA. No applicable.

Table S5. Top 50 highest expressed "*Candidatus* Liberibacter asiaticus" genes in fruit pith.

Gene locus	Gene description	Gene length (bp)	'Guanxi' pomelo		'Shatian' pomelo		'Shatangju' mandarin (TPM)
			TPM	Log_2 fold change	TPM	Log_2 fold change	
CD16_RS03055	outer membrane beta-barrel protein	618	5,146	-1.2	3,700	-1.7	10,103
CD16_RS05110	hypothetical protein	681	3,377	-1.0	2,814	-1.2	5,562
CD16_RS00895	lytic murein transglycosylase	867	2,571	-1.2	1,489	-2.0	4,982
CD16_RS00940	porin	1,056	2,459	-0.4	2,596	-0.3	2,690
CD16_RS01420	prolipoprotein diacylglyceryl transferase	867	2,754	0.0	2,696	0.0	2,283
CD16_RS02245	FAD-binding oxidoreductase	1,422	2,616	0.2	2,474	0.1	1,932
CD16_RS04695	succinate dehydrogenase iron-sulfur subunit	780	2,130	0.0	2,316	0.2	1,754
CD16_RS02000	ParA family protein	798	2,998	0.2	3,282	0.4	2,187
CD16_RS01845	iron-sulfur cluster assembly protein	306	3,084	0.3	2,890	0.2	2,071
CD16_RS02505	septation protein IspZ	615	2,049	-0.2	2,283	-0.1	2,042
CD16_RS00745	4-hydroxy-tetrahydrodipicolinate synthase	879	2,482	-0.2	2,243	-0.3	2,360
CD16_RS00135	phosphoserine transaminase	1,176	2,551	0.0	2,601	0.0	2,212
CD16_RS02145	UMP kinase	729	2,312	-0.1	2,531	0.1	2,025
CD16_RS05415	guanylate kinase	573	3,214	0.5	4,173	0.9	1,900
CD16_RS04255	thymidylate synthase	795	2,192	0.2	1,791	-0.1	1,639
CD16_RS02965	adenylosuccinate synthase	1,299	2,315	0.3	1,919	0.1	1,545
CD16_RS02655	type II toxin-antitoxin system RatA family toxin	459	2,488	0.0	2,649	0.1	2,054
CD16_RS02895	30S ribosomal protein S20	273	2,887	-0.7	1,536	-1.6	4,020
CD16_RS00965	translation initiation factor IF-3	600	2,348	-0.8	2,768	-0.5	3,397
CD16_RS01650	30S ribosomal protein S1	1,731	2,534	-0.2	2,308	-0.3	2,506
CD16_RS01460	50S ribosomal protein L25/general stress protein Ctc	576	1,374	-1.1	887	-1.7	2,486
CD16_RS00550	30S ribosomal protein S11	390	2,314	-0.3	2,418	-0.2	2,449
CD16_RS00825	50S ribosomal protein L13	465	1,558	-0.8	1,206	-1.2	2,338
CD16_RS01465	aspartate--tRNA ligase	1,806	2,884	0.1	2,741	0.0	2,324
CD16_RS01020	50S ribosomal protein L9	501	2,758	0.1	2,586	0.0	2,197
CD16_RS00570	50S ribosomal protein L15	456	3,146	0.4	2,505	0.1	2,071
CD16_RS00830	30S ribosomal protein S9	513	1,174	-1.0	767	-1.6	2,025
CD16_RS00080	50S ribosomal protein L1	696	2,401	0.0	2,537	0.1	2,004

CD16_RS00260	tRNA guanosine(34) transglycosylase Tgt	1,137	2,607	0.4	2,621	0.5	1,638
CD16_RS00660	50S ribosomal protein L4	621	2,159	0.2	2,271	0.3	1,618
CD16_RS00685	30S ribosomal protein S7	471	2,290	0.3	2,250	0.3	1,592
CD16_RS04035	cold-shock protein	237	1,449	-1.7	1,248	-1.9	4,059
CD16_RS01455	CarD family transcriptional regulator	567	1,617	-1.1	1,379	-1.3	2,976
CD16_RS03935	response regulator transcription factor	711	1,494	-0.9	1,609	-0.7	2,307
CD16_RS02925	iron-responsive transcriptional regulator RirA	435	2,055	0.3	2,328	0.5	1,436
CD16_RS05855	DnaB-like helicase C-terminal domain-containing protein	645	4,094	0.2	4,342	0.3	3,002
CD16_RS02890	chromosomal replication initiator protein DnaA	1,509	913	-1.7	718	-2.0	2,541
CD16_RS01325	DNA polymerase III subunit chi	426	2,587	0.2	2,462	0.1	1,942
CD16_RS05395	DNA polymerase	2,028	2,853	0.4	2,581	0.3	1,787
CD16_RS02900	bifunctional DNA-formamidopyrimidine glycosylase/DNA-(apurinic or apyrimidinic site) lyase	870	2,169	0.1	2,626	0.4	1,692
CD16_RS02920	Hsp20 family protein	474	3,337	-1.1	5,004	-0.5	6,012
CD16_RS01515	tRNA (adenosine(37)-N6)-threonylcarbamoyltransferase complex dimerization subunit type 1 TsaB	621	5,071	0.1	5,388	0.2	4,023
CD16_RS03700	chaperonin GroEL	1,656	2,098	-0.6	2,464	-0.4	2,744
CD16_RS01520	NifU family protein	570	2,496	0.0	2,731	0.2	2,101
CD16_RS02830	molecular chaperone DnaK	1,938	1,595	-0.6	1,311	-0.9	2,094
CD16_RS00740	SsrA-binding protein SmpB	480	2,292	0.2	2,145	0.1	1,729
CD16_RS03310	ATP-dependent zinc metalloprotease FtsH	1,980	2,174	0.2	2,051	0.2	1,581
CD16_RS02405	prepilin peptidase	531	1,663	-0.1	2,440	0.5	1,529
CD16_RS02445	ferritin	495	3,777	-1.0	2,577	-1.5	6,200
CD16_RS03555	superoxide dismutase	618	1,651	-0.6	1,159	-1.1	2,151
CD16_RS01625	Bax inhibitor-1/YccA family protein	780	2,487	-0.6	1,865	-1.0	3,224
CD16_RS00725	energy-dependent translational throttle protein EttA	1,620	3,812	0.2	3,678	0.2	2,739

CD16_RS05915	hypothetical protein	351	2,184	-0.3	777	-1.8	2,340
CD16_RS05510	portal protein	1,620	3,030	0.2	2,120	-0.3	2,187
CD16_RS02230	YbaB/EbfC family nucleoid-associated protein	324	2,409	0.2	2,594	0.3	1,807
CD16_RS01775	DciA family protein	486	1,750	-0.1	2,488	0.4	1,632
CD16_RS01695	insulinase family protein	1,275	2,566	0.4	2,343	0.3	1,624
CD16_RS02370	Flp family type IVb pilin	183	5,044	-0.6	7,885	0.0	6,634
CD16_RS04880	hypothetical protein	282	2,343	-2.0	2,797	-1.7	7,849
CD16_RS04890	hypothetical protein	483	2,316	-1.9	2,381	-1.8	7,267
CD16_RS04885	hypothetical protein	327	1,651	-2.1	2,305	-1.6	6,146
CD16_RS04895	hypothetical protein	180	1,026	-2.8	498	-3.8	6,135
CD16_RS02255	hypothetical protein	489	2,082	-1.1	1,597	-1.5	3,788
CD16_RS04385	hypothetical protein	369	1,752	-1.3	1,619	-1.4	3,606
CD16_RS05225	hypothetical protein	465	2,152	-0.7	2,191	-0.7	3,004
CD16_RS05470	hypothetical protein	2,262	3,667	0.1	2,615	-0.3	2,806
CD16_RS05355	hypothetical protein	285	3,731	0.2	3,791	0.3	2,672
CD16_RS05440	hypothetical protein	945	2,664	-0.2	2,252	-0.4	2,540
CD16_RS05680	phosphoglycerate dehydrogenase	1,553	2,817	0.1	2,976	0.2	2,148
CD16_RS03095	hypothetical protein	276	1,889	-0.4	1,349	-0.9	2,105
CD16_RS05485	hypothetical protein	1,773	2,663	0.2	1,657	-0.5	2,012
CD16_RS04525	hypothetical protein	300	2,224	0.3	2,503	0.5	1,555
CD16_RS05585	hypothetical protein	324	1,726	0.3	2,662	1.0	1,179
CD16_RS05580	hypothetical protein	372	374	-0.6	3,684	2.7	489

Table S6. Changes in amino acids in CLas-infected and healthy fruit pith of three cultivars.

Name	'Guanxi' pomelo	'Shatian' pomelo	'Shatangju' mandarin
N-Methyl-L-glutamic acid	12.8	NA	6.3
Thiamine	10.0	NA	NA
Ornithine	9.2	NA	2.2
Agmatine	6.8	NA	1.4
N-Acetyl-L-aspartic acid	3.8	NA	3.0
N6-Acetyl-L-lysine	2.2	NA	1.4
L-Arginine	3.7	2.2	NA
N-Acetyl-L-citrulline	28.6	16.8	4.5
Glycy-L-leucine	2.7	2.4	1.4
D-allo-Isoleucine	2.6	2.7	1.8
N-Acetyl-L-phenylalanine	2.5	2.4	-1.3
L-Lysine	2.2	3.9	NA
2-Amino-5-oxohexanoate	2.1	NA	1.8
1-	1.8	-1.7	NA
L-Histidine	1.7	-1.7	NA
L-Glutamine	1.7	NA	-1.9
Aminoadipic acid	1.6	2.0	NA
Taurine	1.6	2.0	NA
N-Formyl-L-methionine	1.4	1.5	-1.4
D-Phenylalanine	1.4	1.8	NA
Kynurenic acid	1.3	2.3	NA
L-Allothreonine	1.3	NA	NA
L-Serine	-1.3	NA	NA
DL-Glutamate	-1.4	NA	2.0
gamma-Aminobutyric acid	-1.5	NA	NA
alpha-Ketoisovaleric acid	-1.9	NA	-1.1
L-Glutamic gamma-	-3.2	NA	-2.3
S-Ribosyl-L-homocysteine	-7.1	NA	NA
(R)-b-aminoisobutyric acid	NA	4.1	NA
(5-L-Glutamyl)-L-glutamate	NA	2.3	NA
D-Arginine	NA	2.0	3.1
Guanidinosuccinic acid	NA	1.9	NA
L-Methionine	NA	1.9	NA
L-Valine	NA	1.5	1.4
L-Aspartic acid	NA	1.4	-1.6
L-Isoleucine	NA	-2.7	NA
L-Phenylalanine	NA	NA	9.3
L-Tryptophan	NA	NA	5.6
L-Theanine	NA	NA	4.2
D-Asparagine	NA	NA	-1.5
L-Glutamic acid	NA	NA	-1.9
2-Amino-2-deoxy-D-gluconate	NA	NA	-3.4
Phosphoserine	NA	NA	-7.7

NA. No applicable.

Table S7. Changes in sugars in CLas-infected and healthy fruit pith of three cultivars.

Name	‘Guanxi’ pomelo	‘Shatian’ pomelo	‘Shatangju’ mandarin
Raffinose	67.3	NA	NA
Arabitol	27.6	NA	NA
6-Tuliposide A	7.8	NA	NA
Furcatin	5.5	3.8	NA
1-Kestose	5.3	NA	3.9
trans-Zeatin-7-beta-D-glucoside	5.2	2.1	4.5
3'-Ketolactose	4.7	2.5	4.9
Maltotetraose	2.2	NA	NA
D-Lyxose	1.7	NA	2.0
Sedoheptulose	1.6	1.6	NA
Scillabiose	1.5	2.9	NA
Sucrose	-2.0	NA	NA
Cellobiose	-2.2	NA	7.8
Trehalose	-2.2	NA	NA
L-Sorbose	-3.8	-4.8	NA
6-Acetyl-D-glucose	-4.2	NA	NA
Alpha-D-Glucose	NA	2.7	1.7
D-Glucose 1-phosphate	NA	1.6	NA
Chitobiose	NA	1.5	NA
Turanose	NA	-1.9	NA
D-(+)-Glucose	NA	-1.9	NA
L-Erythrulose	NA	-2.0	NA
D-Mannose	NA	-2.1	NA
Melezitose	NA	-2.7	NA
1-O-Feruloyl-beta-D-glucose	NA	-2.8	NA
Maltotriose	NA	NA	126.1
D-Glucose	NA	NA	5.0
Fructose 1,6-bisphosphate	NA	NA	-2.1

NA. No applicable.

Table S8. Changes in organic acids in CLas-infected and healthy fruit pith of three cultivars.

Name	‘Guanxi’ pomelo	‘Shatian’ pomelo	‘Shatangju’ mandarin
6-Hydroxynicotinic acid	14.2	NA	NA
Hydroxypyruvic acid	3.5	NA	1.8
Uric acid	2.3	NA	-1.6
3-Methyl-2-oxovaleric acid	2.1	NA	-1.2
4-(beta-D-Glucosyloxy) benzoate	1.9	NA	NA
Tartaric acid	1.9	-2.6	NA
Acetylphosphate	1.8	NA	NA
Citric acid	1.5	NA	NA
L-Malic acid	1.3	-1.6	-1.5
Formylanthranilic acid	1.5	NA	NA
Ketoleucine	-1.8	NA	1.9
5-Amino-2-oxopentanoic acid	-2.9	NA	NA
Gulonic acid	-3.6	NA	NA
gamma-Glutamylalanine	NA	2.3	NA
4-Hydroxybenzoic acid	NA	1.7	NA
Isocitric acid	NA	-2.0	190.5
beta-Citryl-L-glutamate	NA	-2.9	NA
Gentisic acid	NA	NA	4.6
Diaminopimelic acid	NA	NA	1.8
L-2,4-diaminobutyric acid	NA	NA	1.5
Oxoadipic acid	NA	NA	1.5
Fumaric acid	NA	NA	-1.6

NA. No applicable.

Table S9. Changes in flavonoids in CLas-infected and healthy fruit pith of three cultivars.

Name	'Guanxi' pomelo	'Shatian' pomelo	'Shatangju' mandarin
Cyanidin 3-rutinoside	72.4	25.0	2.8
Astilbin	23.3	NA	NA
Hesperidin	7.0	11.6	3.9
Vitexin	3.2	NA	NA
Cyanin	2.6	3.1	NA
Naringin	2.2	NA	NA
Flavonol 7-O-beta-D-glucoside	2.1	NA	NA
Tangeritin	1.5	NA	NA
Sakuranetin	1.5	NA	NA
Pinocembrin 7-rhamnosylglucoside	1.5	1.5	NA
Isovitexin	-2.1	NA	-1.8
Acerosin	-2.6	NA	NA
(2S)-Liquiritigenin	-12.5	NA	NA
Apigenin	-25.0	NA	2.2
Isoflavone 7-O-beta-D-glucoside	NA	19.3	NA
Hesperetin 7-O-glucoside	NA	3.9	-5.3
Leucopelargonidin	NA	1.5	NA
Glycitein	NA	-2.2	-2.6
Isovitexin 2'-O-beta-D-glucoside	NA	-5.3	NA
Hesperetin 7-neohesperidoside	NA	-5.3	NA
8-C-Glucosylnaringenin	NA	NA	4.5
Naringenin 7-O-beta-D-glucoside	NA	NA	-1.8
Poncirin	NA	NA	-2.1
Hesperetin	NA	NA	-2.1

NA. No applicable.

Table S10. Changes in terpenoids in CLas-infected and healthy fruit pith of three cultivars.

Name	‘Guanxi’ pomelo	‘Shatian’ pomelo	‘Shatangju’ mandarin
Lamioside	14.1	NA	NA
Secologanin	8.8	6.5	NA
Quadrone	4.8	NA	3.9
Gardenoside	3.5	1.8	14.9
Limonin	2.7	NA	-2.5
Capsidiol	2.2	NA	-1.4
Xanthoxic acid	2.0	NA	NA
Obacunone	1.9	1.2	NA
Parthenin	1.8	2.0	NA
Harpagoside	1.8	NA	NA
Menthone	1.5	NA	NA
Caryophyllene alpha-oxide	1.5	NA	NA
Perillyl alcohol	-2.0	NA	-1.9
Nomilin	NA	5.5	NA
Bilobalide A	NA	5.0	NA
Polygodial	NA	4.4	NA
p-Cymene	NA	2.7	NA
Qing Hau Sau	NA	1.4	1.6

NA. No applicable.

Table S11. Primer sets used in this study.

Target gene ID	Product	Primer	Sequence (5' - 3')	Size (bp)	Reference
L22532.1	16S rRNA	CLas_4G<br>HLBr	AGTCGAGCGCGTATGCGAAT<br>GCGTTATCCCGTAGAAAAAGGTAG	78	Bao et al., 2020
CD16_RS04295	ribonucleotide-diphosphate reductase subunit beta	04295-F<br>04295-R	TAAAATACGGATGGGCATGG<br>TTCCACAGTGCAAGATCGTC	97	This study
CD16_RS05750	DEAD/DEAH box helicase	05750-F<br>05750-R	GCCATGGCTTAAATCTCCAA<br>CCAATGCGTTCAATCATCTG	97	This study
CD16_RS00025	hypothetical protein	00025-F<br>00025-R	TCGCAATCGAAGGGTAAAGT<br>ATAGCCATCCCATGCAAAAG	99	This study
CD16_RS02255	hypothetical protein	02255-F<br>02255-R	TGTGTTGAGTGTCCGTCTCC<br>GGGACATAAACCCTTTGACG	93	This study
CD16_RS04385	hypothetical protein	04385-F<br>04385-R	AATAAACCCGTGCGGCATAG<br>TTTTCATTTTCCGCAGACAG	90	This study
CD16_RS03985	NADP-dependent isocitrate dehydrogenase	03985-F<br>03985-R	TTTTTGATTCGCCTGGAAGT<br>TGCTAACGCGTATTGCATTG	98	This study
CD16_RS04710	malate dehydrogenase	04710-F<br>04710-R	GTGAAGAGAACTCGCGAAGG<br>GGCTATGGCAGAAGAAGCAG	90	This study
CD16_RS05110	hypothetical protein	05110-F<br>05110-R	ATTGGTGTGGGGATTGAAAA<br>ACATCCCAAGGCTGGTCATA	92	This study
CD16_RS03055	outer membrane beta-barrel protein	03055-F<br>03055-R	CTCAAGCAGCTGATCCTGTG<br>GTGGCGAATCGGAACATATC	92	This study
CD16_RS02445	ferritin	02445-F<br>02445-R	TGCAATGGAAGAACACTCTCA<br>TAATCACAGGCGCTTCAATG	98	This study
